# Supplementary material for: 21-nt phasiRNAs direct target mRNA cleavage in rice male germ cells
Source: Nat Commun. 2020 Oct 15;11:5191. doi: 10.1038/s41467-020-19034-y (PMC7562718; doi:10.1038/s41467-020-19034-y)
Supplement: Supplementary file 1 — Supplementary Information [file 41467_2020_19034_MOESM1_ESM.pdf]

Supplementary Information

Supplementary Figures

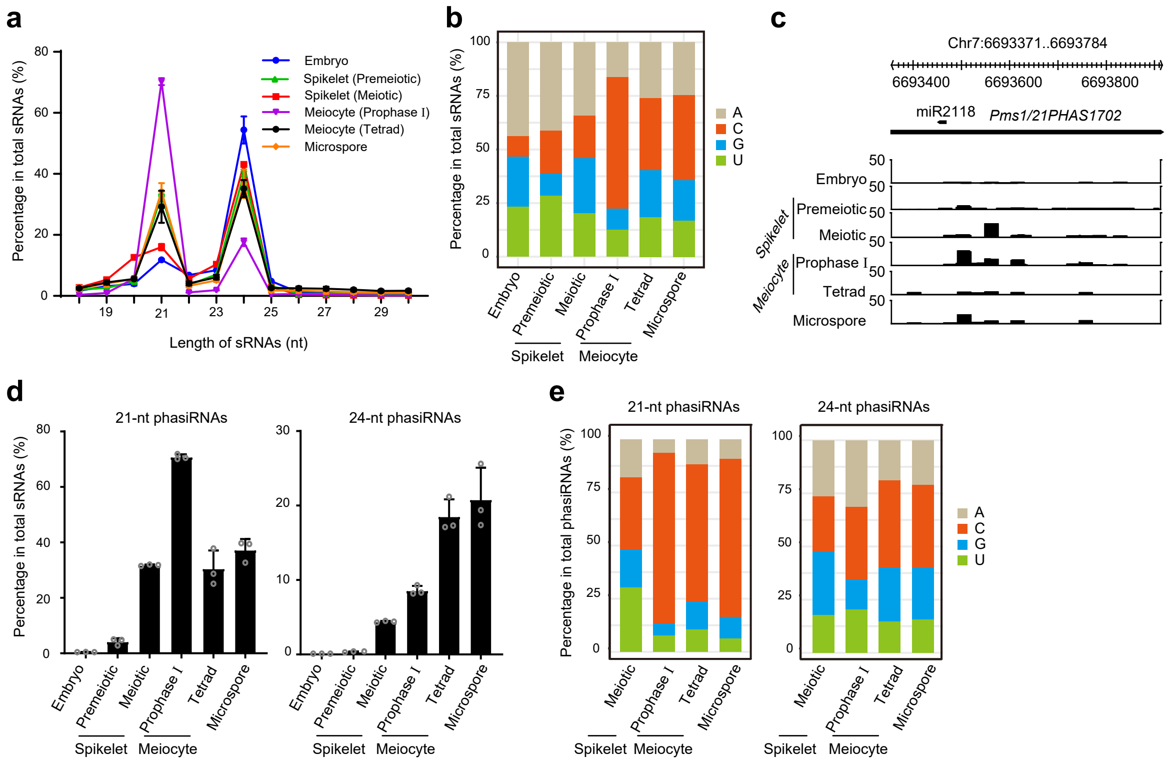

Supplementary Fig. 1. Profiling of phasiRNAs in rice spikelets and purified germ cells. a, Length distribution of genome-matched sRNAs in embryos, spikelets, meiocytes and microspores. **b**, 5' terminal nucleotide frequency of genome-matched sRNAs in embryos, spikelets, meiocytes and microspores. **c**, Genome browser views of 21-nt phasiRNA signals at the *Pms1* locus in embryos, spikelets, meiocytes and microspores. The y-axis represents sRNA abundance (RPM). **d**, Percentages of 21- and 24-nt phasiRNAs in total genome-matched sRNAs in embryos, spikelets, meiocytes and microspores. The error bars represent standard deviation of three biological replicates. **e**, 5' terminal nucleotide frequency of 21- and 24-nt phasiRNAs in meiotic spikelets, meiocytes and microspores.

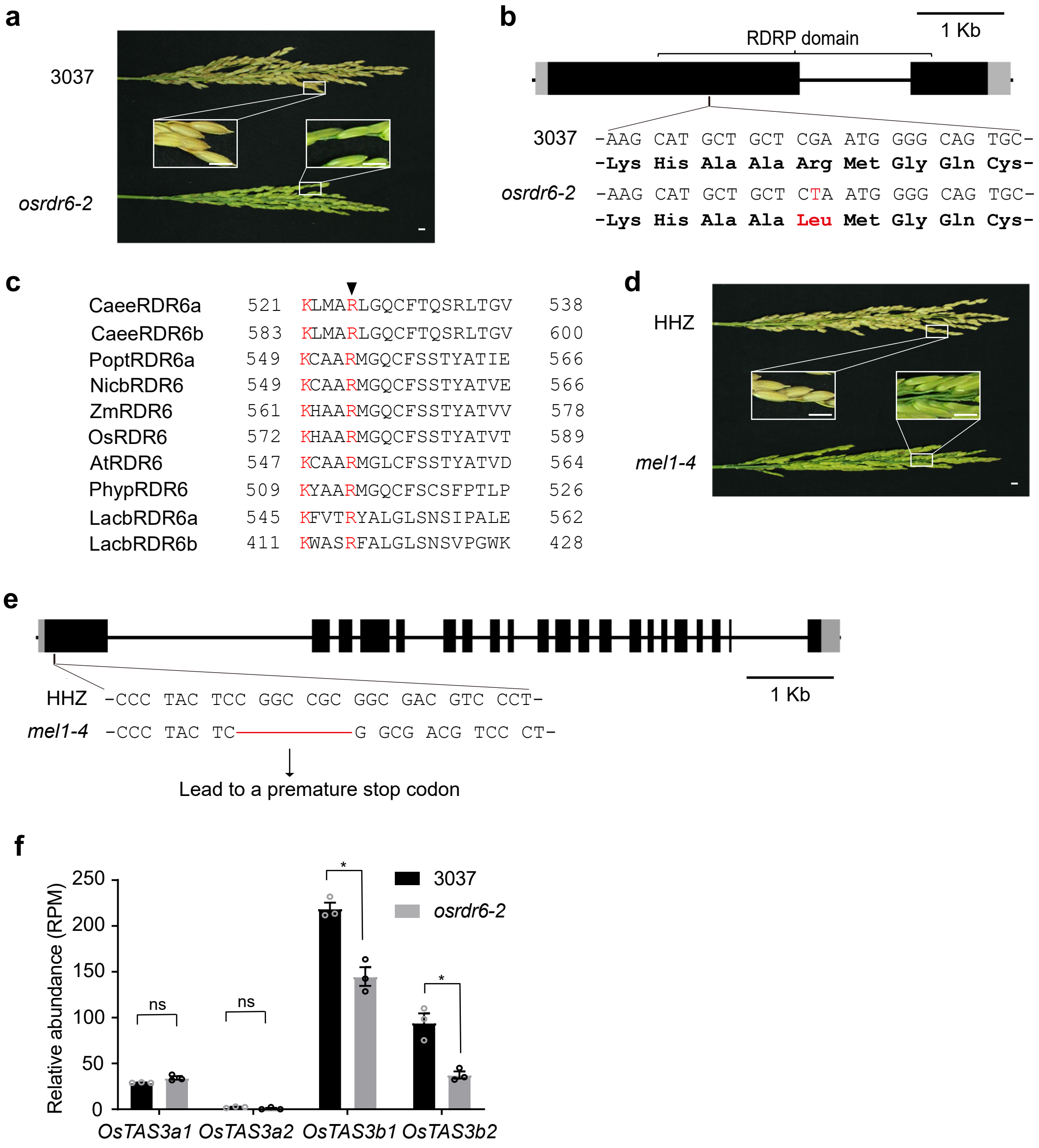

Supplementary Fig. 2. Characterization of the *osrdr6-2* and *mel1-4* mutants.

a, Panicles of *osrdr6-2* and 3037 rice plants at the grain-filling stage. Note that the seeds of *osrdr6-2* are unfilled and green. Scale bars, 0.5 cm. **b**, Gene structures of *OsRDR6*. Exons are indicated by boxes and introns are indicated by lines. *osrdr6-2* carries point mutation that leads to an amino acid change. **c**, Alignment of partial RDR6 protein sequences. The sequences were aligned with ClustalX. Conserved residues are shown in red. **d**, Panicles of *mel1-4* and HHZ rice plants at the grain-filling stage. Note that the seeds of *mel1-4* are unfilled and green. Scale bars, 0.5 cm. **e**, Gene structures of *MEL1*. Exons are

indicated by boxes and introns are indicated by lines. *mel1-4* has a deletion mutation that results in a premature stop codon. **f,** Relative accumulation levels of *TAS3*-derived tasiRNAs in early prophase I meiocytes of 3037 and *osrdr6-2*. The error bars represent standard deviation of three biological replicates. Asterisks indicate significant differences between *osrdr6-2* and the corresponding wild-type 3037 plants. *, Student's t test，*p*-value ≤ 0.05.

a

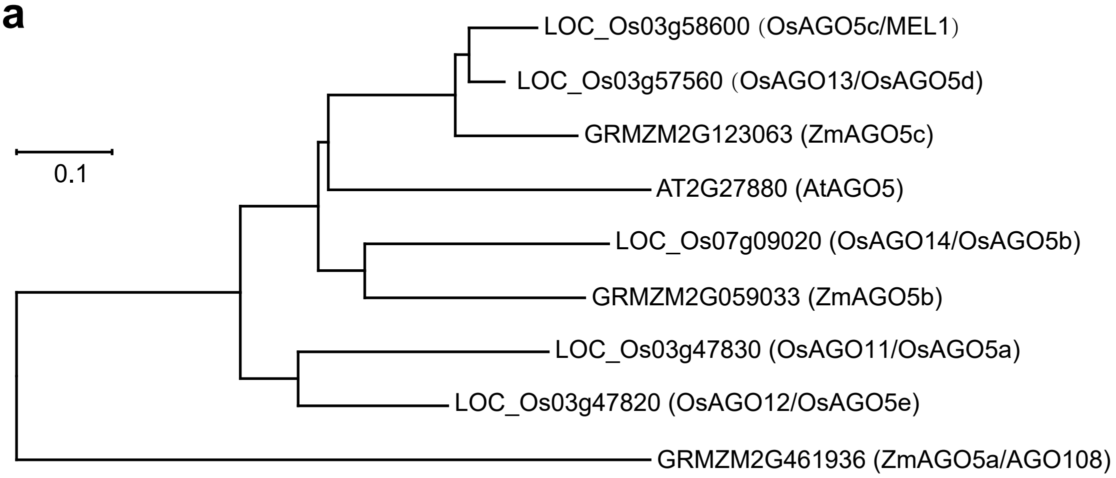

b

AtAGO1	755	PTIIFGADVTHP......IIFYRDGVSEG......PAYYAHLAAFRA	994
OsAGO5a	603	PTMIFGADVTHP......IIFYRDGVSEG......PVYYAHLAASRA	843
OsAGO14/OsAGO5b	751	TTIIFGADVTHP......IIFYRDGVSDG......PAYYAHLAAFRA	1004
MEL1/OsAGO5c	773	PTIIFGADVTHP......IIFYRDGVSEG......PAYYAHLAAFRA	1011
OsAGO5d	402	-------LMSHT......IIFYRDGVSEG......PAYYSHLAASHA	633
OsAGO5e	779	PTIVFGADVTHP......IIFYRDGISEG......PAYYAHLGAFRA	1016

Supplementary Fig. 3. AGO5 subclade proteins in rice, maize and *Arabidopsis*. a, Phylogenetic analysis of AGO5 proteins in rice, maize and *Arabidopsis*. The neighbor joining tree was constructed using the MEGAX software. At, *Arabidopsis thaliana*; Os, *Oryza sativa*; Zm, *Zea mays*. Scale bars, 0.1 substitutions per amino acid position. **b**, Alignment of AGO5 protein sequences. The sequences were aligned with ClustalX. Residues forming the catalytic DDH motif are shown in red.

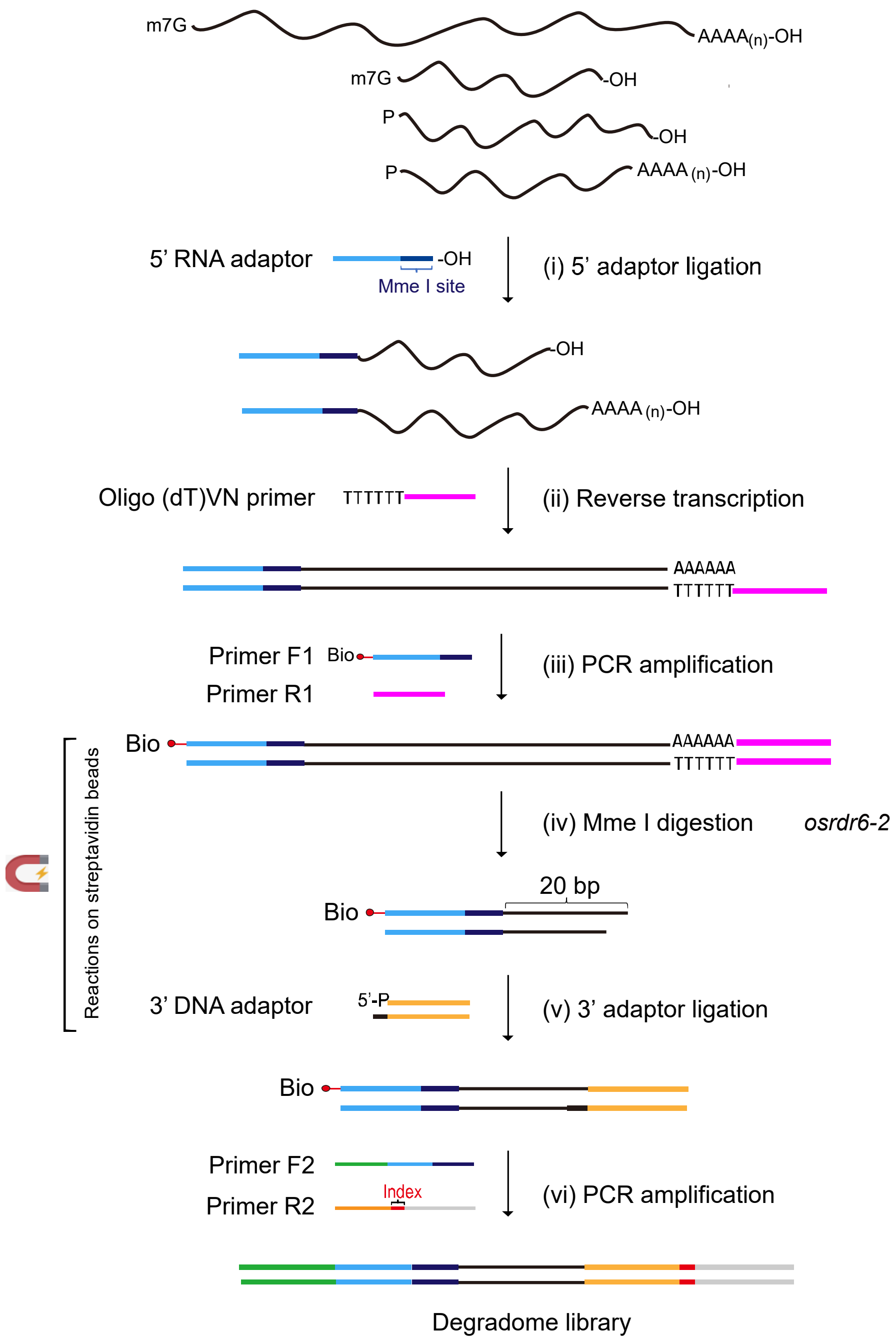

Supplementary Fig. 4. Schematic depiction of low-input degradome library construction. Construction of a degradome library for low-input RNAs includes the following steps: (i) RNA with 5’-monophophates group in total RNA is ligated to the 5’ RNA adaptor; (ii) the ligated product with 3’ poly-A is reverse-transcribed with Oligo-dT$_{30}$VN primer; (iii) the reverse transcription product is purified and amplified with biotin-labelled primer F1 and primer R1; (iv) the PCR product was purified and digested with MmeI; (v) the digested product is purified and ligated to the 3’ DNA adaptor; (vi) the final ligated product is purified and amplified with primer F2 and primer R2.

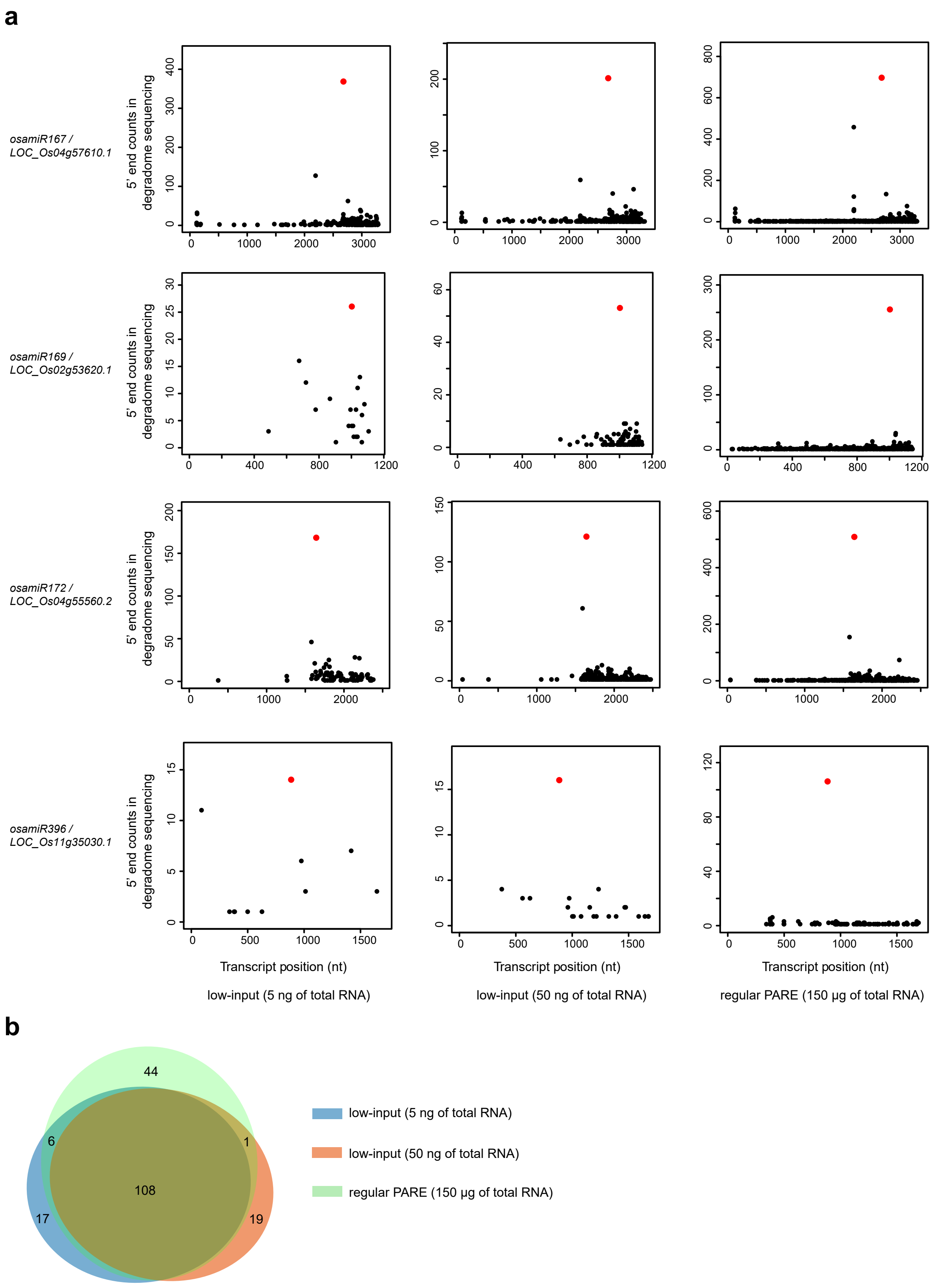

Supplementary Fig. 5. Comparison of the specificity of the low-input and regular PARE protocols. a, Plots showing the distribution of the *degradome* tags *along miRNA targets*. The x-axis represents the nucleotide position of each target mRNA (5'-3'). The y-axis represents the

relative frequency of degradome tags. The red dot represents degradome tag at +1 position of miRNA-guided cleavage site. **b**, Venn diagram showing the overlap between miRNA targets identified by the low-input protocol (with 5 ng and 50 ng of total RNA) and the regular PARE protocol (with 150 μg of total RNA).

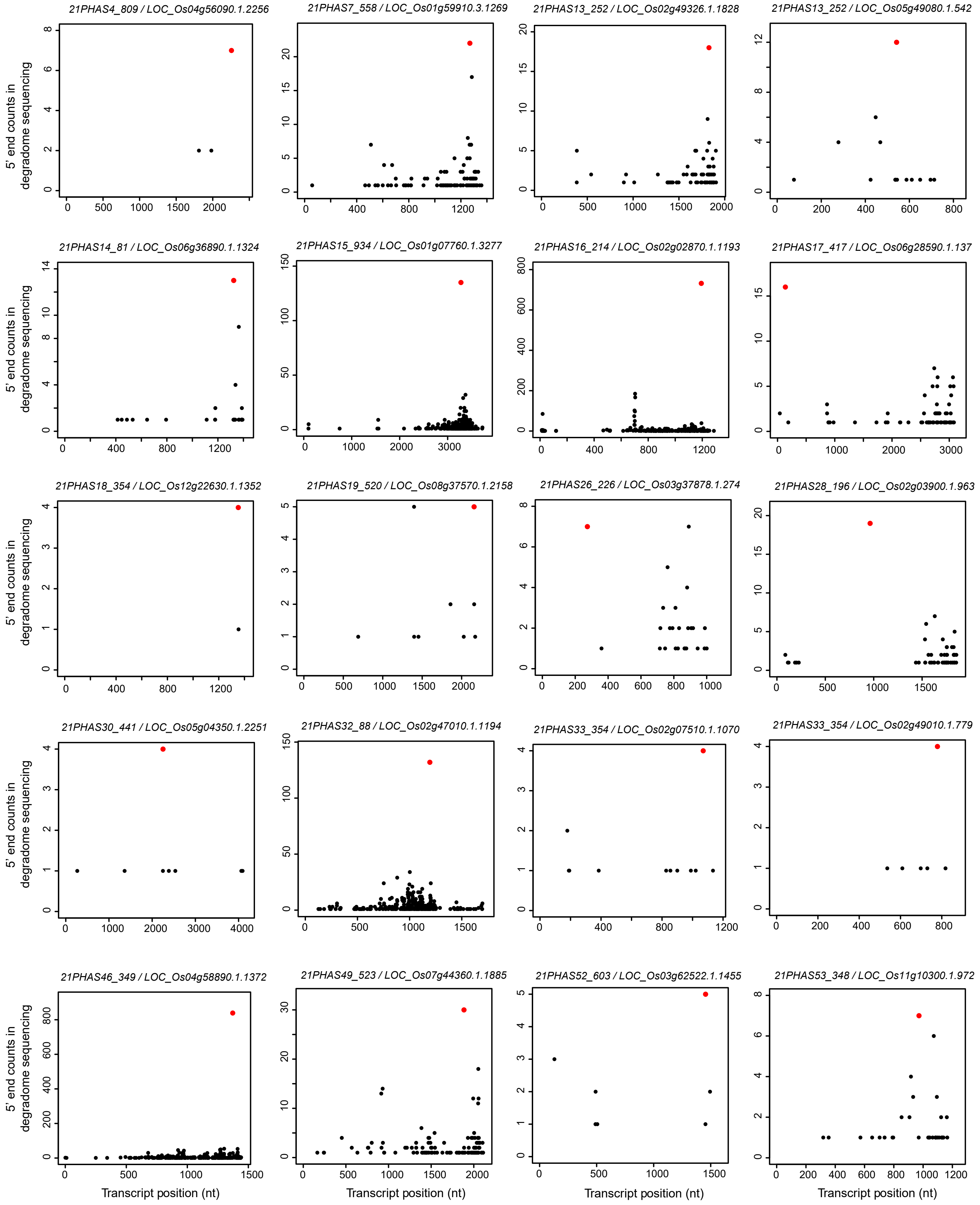
21PHAS4_809 / LOC_Os04g56090.1.2256
21PHAS7_558 / LOC_Os01g59910.3.1269
21PHAS13_252 / LOC_Os02g49326.1.1828
21PHAS13_252 / LOC_Os05g49080.1.542
21PHAS14_81 / LOC_Os06g36890.1.1324
21PHAS15_934 / LOC_Os01g07760.1.3277
21PHAS16_214 / LOC_Os02g02870.1.1193
21PHAS17_417 / LOC_Os06g28590.1.137
21PHAS18_354 / LOC_Os12g22630.1.1352
21PHAS19_520 / LOC_Os08g37570.1.2158
21PHAS26_226 / LOC_Os03g37878.1.274
21PHAS28_196 / LOC_Os02g03900.1.963
21PHAS30_441 / LOC_Os05g04350.1.2251
21PHAS32_88 / LOC_Os02g47010.1.1194
21PHAS33_354 / LOC_Os02g07510.1.1070
21PHAS33_354 / LOC_Os02g49010.1.779
21PHAS46_349 / LOC_Os04g58890.1.1372
21PHAS49_523 / LOC_Os07g44360.1.1885
21PHAS52_603 / LOC_Os03g62522.1.1455
21PHAS53_348 / LOC_Os11g10300.1.972
5' end counts in degradome sequencing
Transcript position (nt)

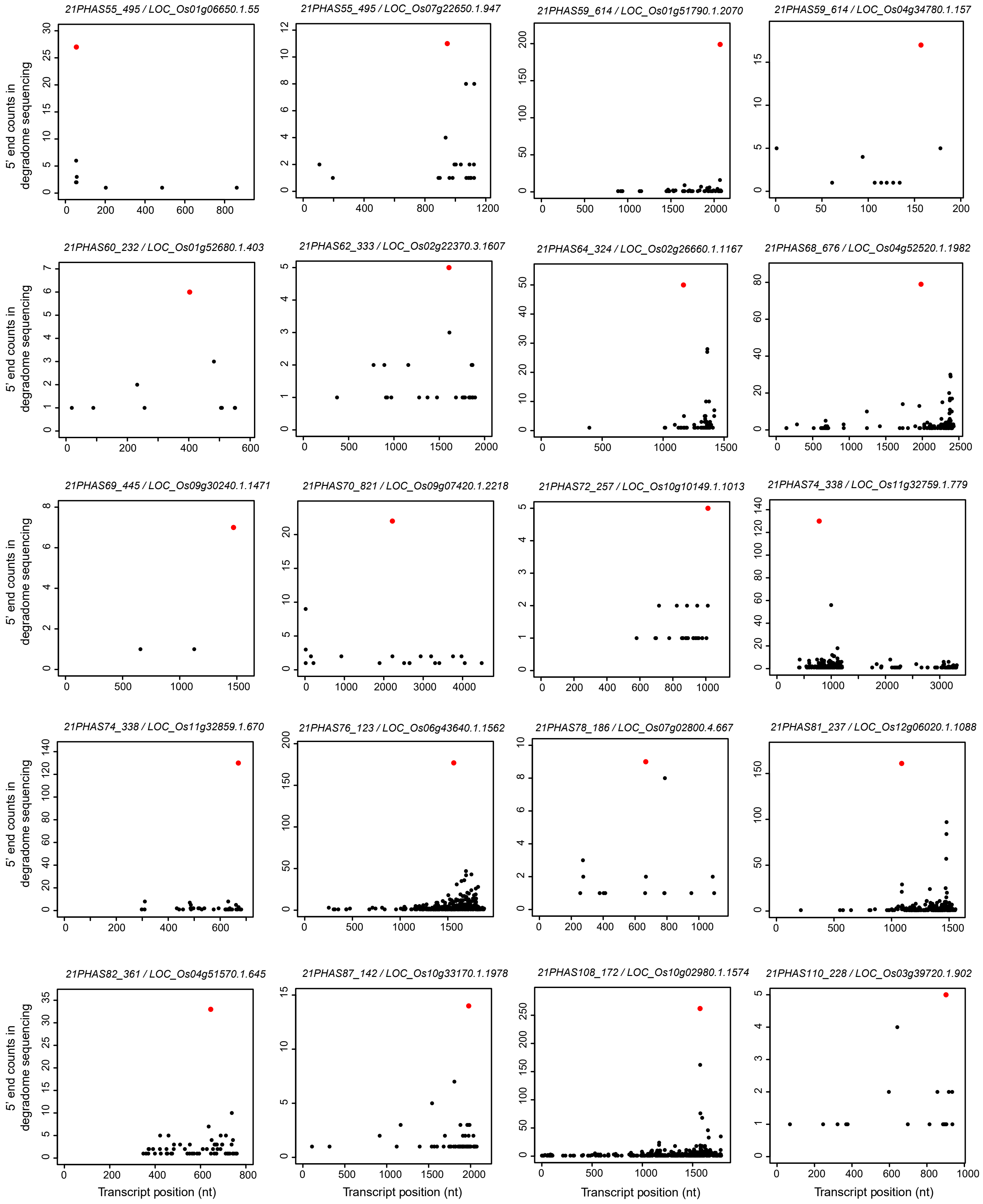

21PHAS55_495 / LOC_Os01g06650.1.55
21PHAS55_495 / LOC_Os07g22650.1.947
21PHAS59_614 / LOC_Os01g51790.1.2070
21PHAS59_614 / LOC_Os04g34780.1.157
21PHAS60_232 / LOC_Os01g52680.1.403
21PHAS62_333 / LOC_Os02g22370.3.1607
21PHAS64_324 / LOC_Os02g26660.1.1167
21PHAS68_676 / LOC_Os04g52520.1.1982
21PHAS69_445 / LOC_Os09g30240.1.1471
21PHAS70_821 / LOC_Os09g07420.1.2218
21PHAS72_257 / LOC_Os10g10149.1.1013
21PHAS74_338 / LOC_Os11g32759.1.779
21PHAS74_338 / LOC_Os11g32859.1.670
21PHAS76_123 / LOC_Os06g43640.1.1562
21PHAS78_186 / LOC_Os07g02800.4.667
21PHAS81_237 / LOC_Os12g06020.1.1088
21PHAS82_361 / LOC_Os04g51570.1.645
21PHAS87_142 / LOC_Os10g33170.1.1978
21PHAS108_172 / LOC_Os10g02980.1.1574
21PHAS110_228 / LOC_Os03g39720.1.902
5' end counts in degradome sequencing
Transcript position (nt)

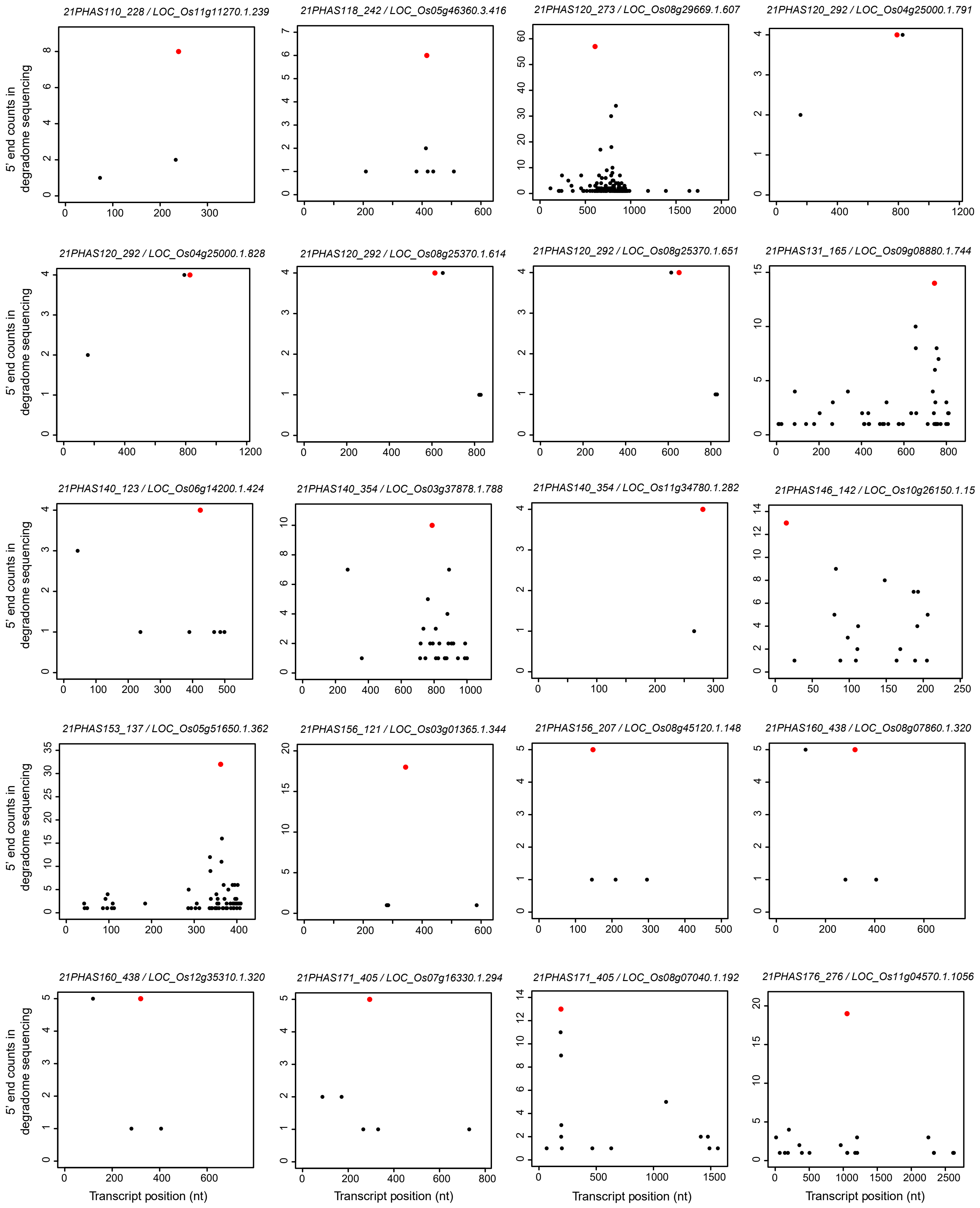
21PHAS110_228 / LOC_Os11g11270.1.239
21PHAS118_242 / LOC_Os05g46360.3.416
21PHAS120_273 / LOC_Os08g29669.1.607
21PHAS120_292 / LOC_Os04g25000.1.791
21PHAS120_292 / LOC_Os04g25000.1.828
21PHAS120_292 / LOC_Os08g25370.1.614
21PHAS120_292 / LOC_Os08g25370.1.651
21PHAS131_165 / LOC_Os09g08880.1.744
21PHAS140_123 / LOC_Os06g14200.1.424
21PHAS140_354 / LOC_Os03g37878.1.788
21PHAS140_354 / LOC_Os11g34780.1.282
21PHAS146_142 / LOC_Os10g26150.1.15
21PHAS153_137 / LOC_Os05g51650.1.362
21PHAS156_121 / LOC_Os03g01365.1.344
21PHAS156_207 / LOC_Os08g45120.1.148
21PHAS160_438 / LOC_Os08g07860.1.320
21PHAS160_438 / LOC_Os12g35310.1.320
21PHAS171_405 / LOC_Os07g16330.1.294
21PHAS171_405 / LOC_Os08g07040.1.192
21PHAS176_276 / LOC_Os11g04570.1.1056
5' end counts in degradome sequencing
Transcript position (nt)

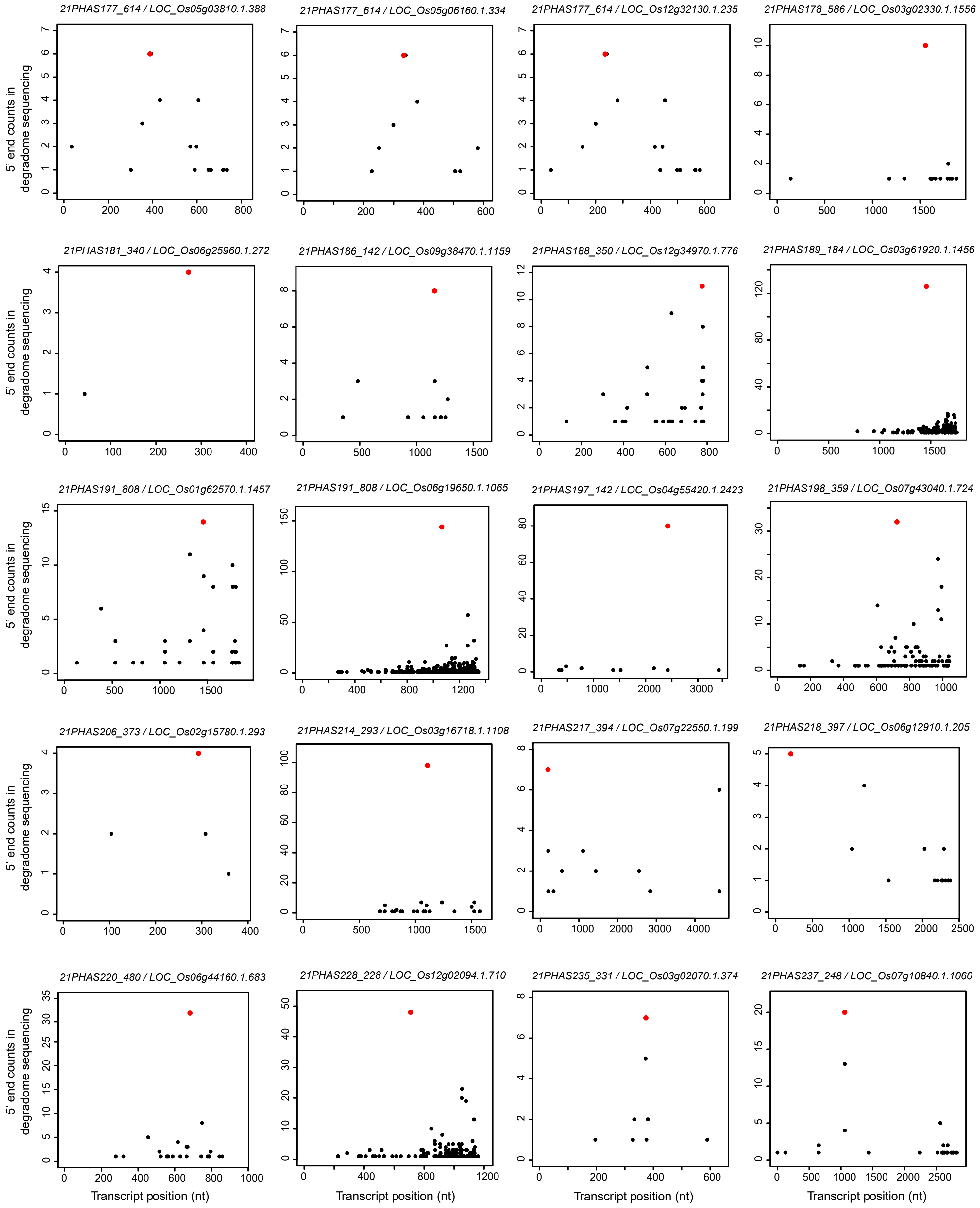

21PHAS177_614 / LOC_Os05g03810.1.388
21PHAS177_614 / LOC_Os05g06160.1.334
21PHAS177_614 / LOC_Os12g32130.1.235
21PHAS178_586 / LOC_Os03g02330.1.1556
21PHAS181_340 / LOC_Os06g25960.1.272
21PHAS186_142 / LOC_Os09g38470.1.1159
21PHAS188_350 / LOC_Os12g34970.1.776
21PHAS189_184 / LOC_Os03g61920.1.1456
21PHAS191_808 / LOC_Os01g62570.1.1457
21PHAS191_808 / LOC_Os06g19650.1.1065
21PHAS197_142 / LOC_Os04g55420.1.2423
21PHAS198_359 / LOC_Os07g43040.1.724
21PHAS206_373 / LOC_Os02g15780.1.293
21PHAS214_293 / LOC_Os03g16718.1.1108
21PHAS217_394 / LOC_Os07g22550.1.199
21PHAS218_397 / LOC_Os06g12910.1.205
21PHAS220_480 / LOC_Os06g44160.1.683
21PHAS228_228 / LOC_Os12g02094.1.710
21PHAS235_331 / LOC_Os03g02070.1.374
21PHAS237_248 / LOC_Os07g10840.1.1060
5' end counts in degradome sequencing
Transcript position (nt)

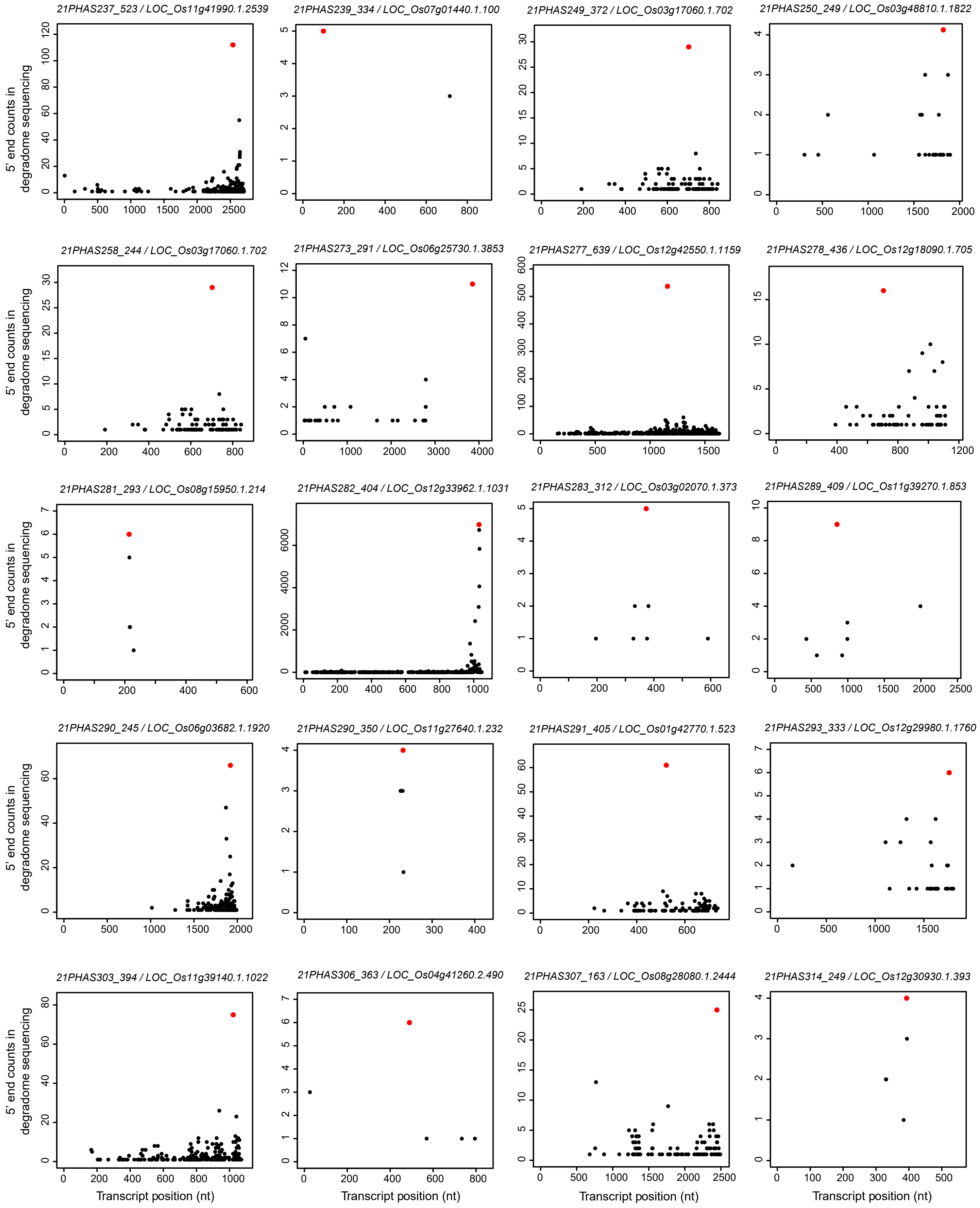
21PHAS237_523 / LOC_Os11g41990.1.2539
21PHAS239_334 / LOC_Os07g01440.1.100
21PHAS249_372 / LOC_Os03g17060.1.702
21PHAS250_249 / LOC_Os03g48810.1.1822
21PHAS258_244 / LOC_Os03g17060.1.702
21PHAS273_291 / LOC_Os06g25730.1.3853
21PHAS277_639 / LOC_Os12g42550.1.1159
21PHAS278_436 / LOC_Os12g18090.1.705
21PHAS281_293 / LOC_Os08g15950.1.214
21PHAS282_404 / LOC_Os12g33962.1.1031
21PHAS283_312 / LOC_Os03g02070.1.373
21PHAS289_409 / LOC_Os11g39270.1.853
21PHAS290_245 / LOC_Os06g03682.1.1920
21PHAS290_350 / LOC_Os11g27640.1.232
21PHAS291_405 / LOC_Os01g42770.1.523
21PHAS293_333 / LOC_Os12g29980.1.1760
21PHAS303_394 / LOC_Os11g39140.1.1022
21PHAS306_363 / LOC_Os04g41260.2.490
21PHAS307_163 / LOC_Os08g28080.1.2444
21PHAS314_249 / LOC_Os12g30930.1.393
5' end counts in degradome sequencing
Transcript position (nt)

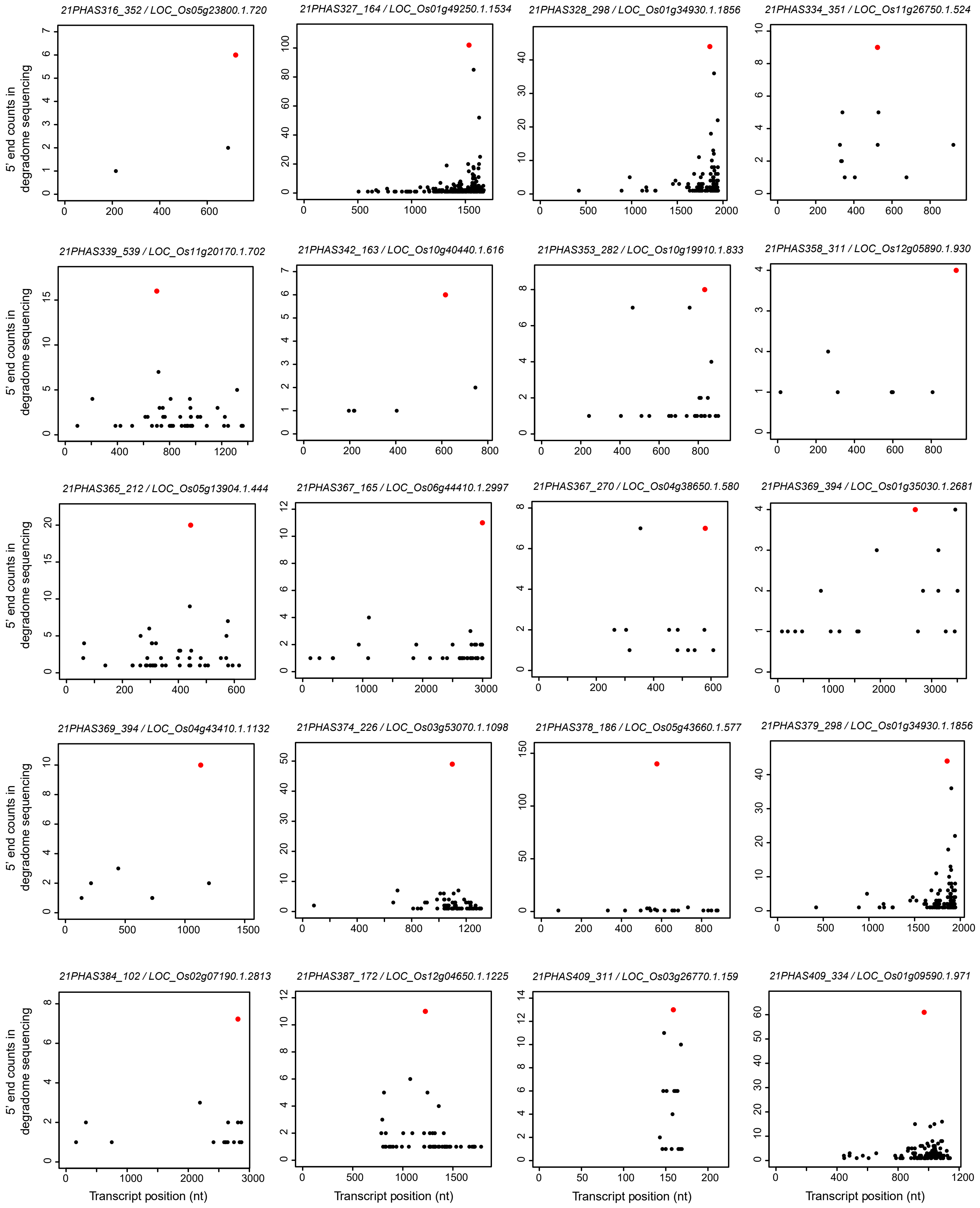
21PHAS316_352 / LOC_Os05g23800.1.720
21PHAS327_164 / LOC_Os01g49250.1.1534
21PHAS328_298 / LOC_Os01g34930.1.1856
21PHAS334_351 / LOC_Os11g26750.1.524
21PHAS339_539 / LOC_Os11g20170.1.702
21PHAS342_163 / LOC_Os10g40440.1.616
21PHAS353_282 / LOC_Os10g19910.1.833
21PHAS358_311 / LOC_Os12g05890.1.930
21PHAS365_212 / LOC_Os05g13904.1.444
21PHAS367_165 / LOC_Os06g44410.1.2997
21PHAS367_270 / LOC_Os04g38650.1.580
21PHAS369_394 / LOC_Os01g35030.1.2681
21PHAS369_394 / LOC_Os04g43410.1.1132
21PHAS374_226 / LOC_Os03g53070.1.1098
21PHAS378_186 / LOC_Os05g43660.1.577
21PHAS379_298 / LOC_Os01g34930.1.1856
21PHAS384_102 / LOC_Os02g07190.1.2813
21PHAS387_172 / LOC_Os12g04650.1.1225
21PHAS409_311 / LOC_Os03g26770.1.159
21PHAS409_334 / LOC_Os01g09590.1.971
5' end counts in degradome sequencing
Transcript position (nt)

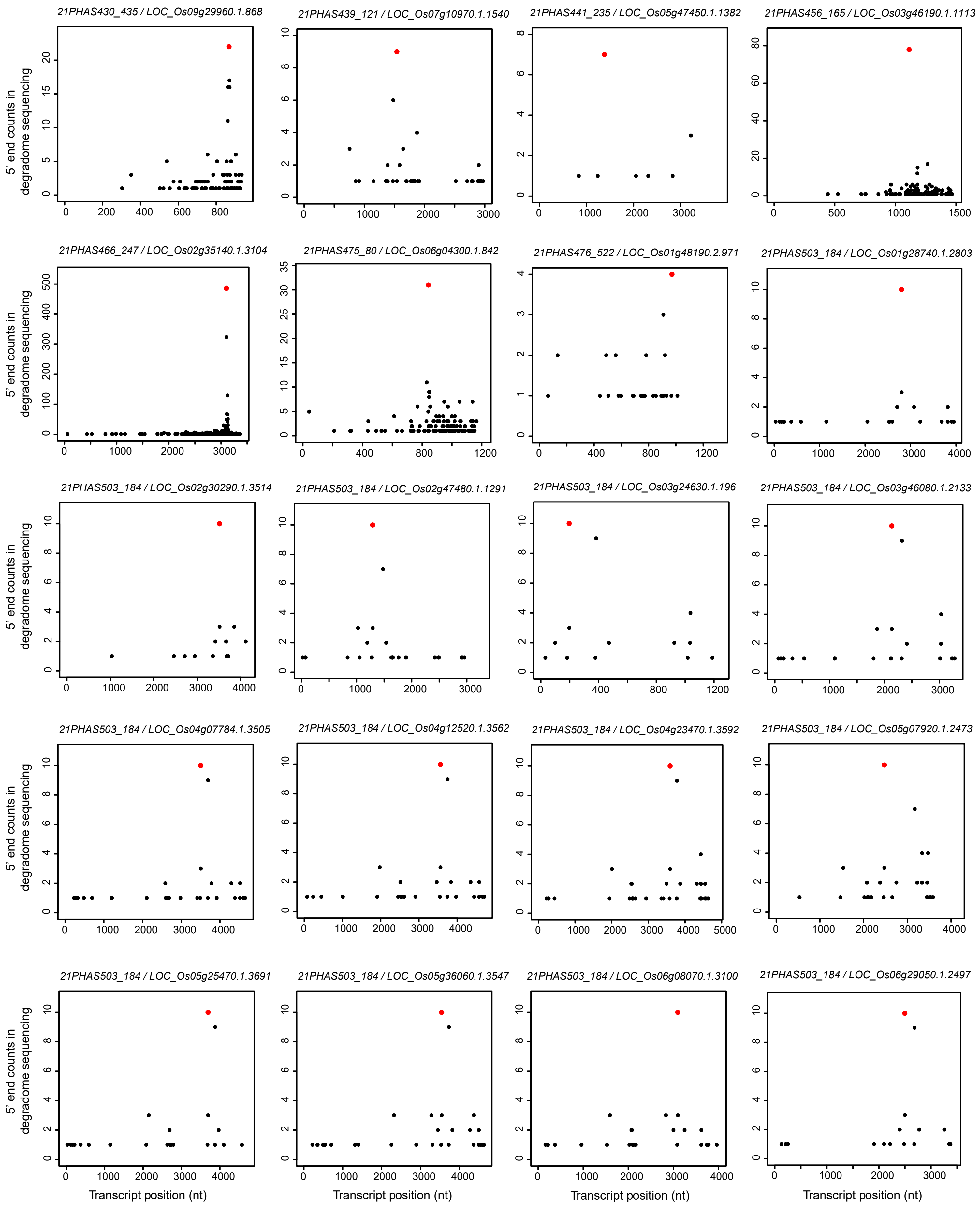

21PHAS430_435 / LOC_Os09g29960.1.868
21PHAS439_121 / LOC_Os07g10970.1.1540
21PHAS441_235 / LOC_Os05g47450.1.1382
21PHAS456_165 / LOC_Os03g46190.1.1113
21PHAS466_247 / LOC_Os02g35140.1.3104
21PHAS475_80 / LOC_Os06g04300.1.842
21PHAS476_522 / LOC_Os01g48190.2.971
21PHAS503_184 / LOC_Os01g28740.1.2803
21PHAS503_184 / LOC_Os02g30290.1.3514
21PHAS503_184 / LOC_Os02g47480.1.1291
21PHAS503_184 / LOC_Os03g24630.1.196
21PHAS503_184 / LOC_Os03g46080.1.2133
21PHAS503_184 / LOC_Os04g07784.1.3505
21PHAS503_184 / LOC_Os04g12520.1.3562
21PHAS503_184 / LOC_Os04g23470.1.3592
21PHAS503_184 / LOC_Os05g07920.1.2473
21PHAS503_184 / LOC_Os05g25470.1.3691
21PHAS503_184 / LOC_Os05g36060.1.3547
21PHAS503_184 / LOC_Os06g08070.1.3100
21PHAS503_184 / LOC_Os06g29050.1.2497
5' end counts in degradome sequencing
Transcript position (nt)

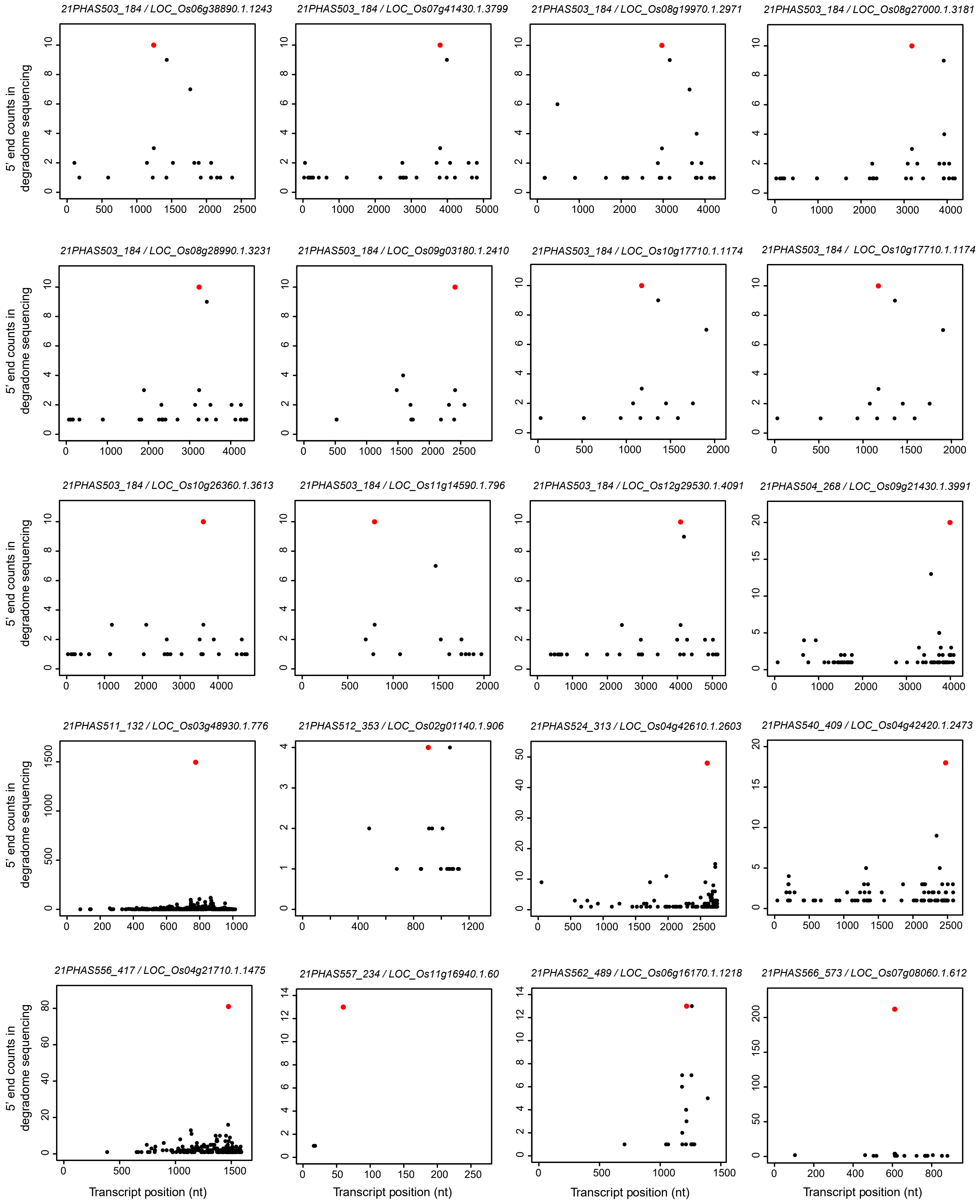

21PHAS503_184 / LOC_Os06g38890.1.1243
21PHAS503_184 / LOC_Os07g41430.1.3799
21PHAS503_184 / LOC_Os08g19970.1.2971
21PHAS503_184 / LOC_Os08g27000.1.3181
21PHAS503_184 / LOC_Os08g28990.1.3231
21PHAS503_184 / LOC_Os09g03180.1.2410
21PHAS503_184 / LOC_Os10g17710.1.1174
21PHAS503_184 / LOC_Os10g17710.1.1174
21PHAS503_184 / LOC_Os10g26360.1.3613
21PHAS503_184 / LOC_Os11g14590.1.796
21PHAS503_184 / LOC_Os12g29530.1.4091
21PHAS504_268 / LOC_Os09g21430.1.3991
21PHAS511_132 / LOC_Os03g48930.1.776
21PHAS512_353 / LOC_Os02g01140.1.906
21PHAS524_313 / LOC_Os04g42610.1.2603
21PHAS540_409 / LOC_Os04g42420.1.2473
21PHAS556_417 / LOC_Os04g21710.1.1475
21PHAS557_234 / LOC_Os11g16940.1.60
21PHAS562_489 / LOC_Os06g16170.1.1218
21PHAS566_573 / LOC_Os07g08060.1.612
5' end counts in degradome sequencing
Transcript position (nt)

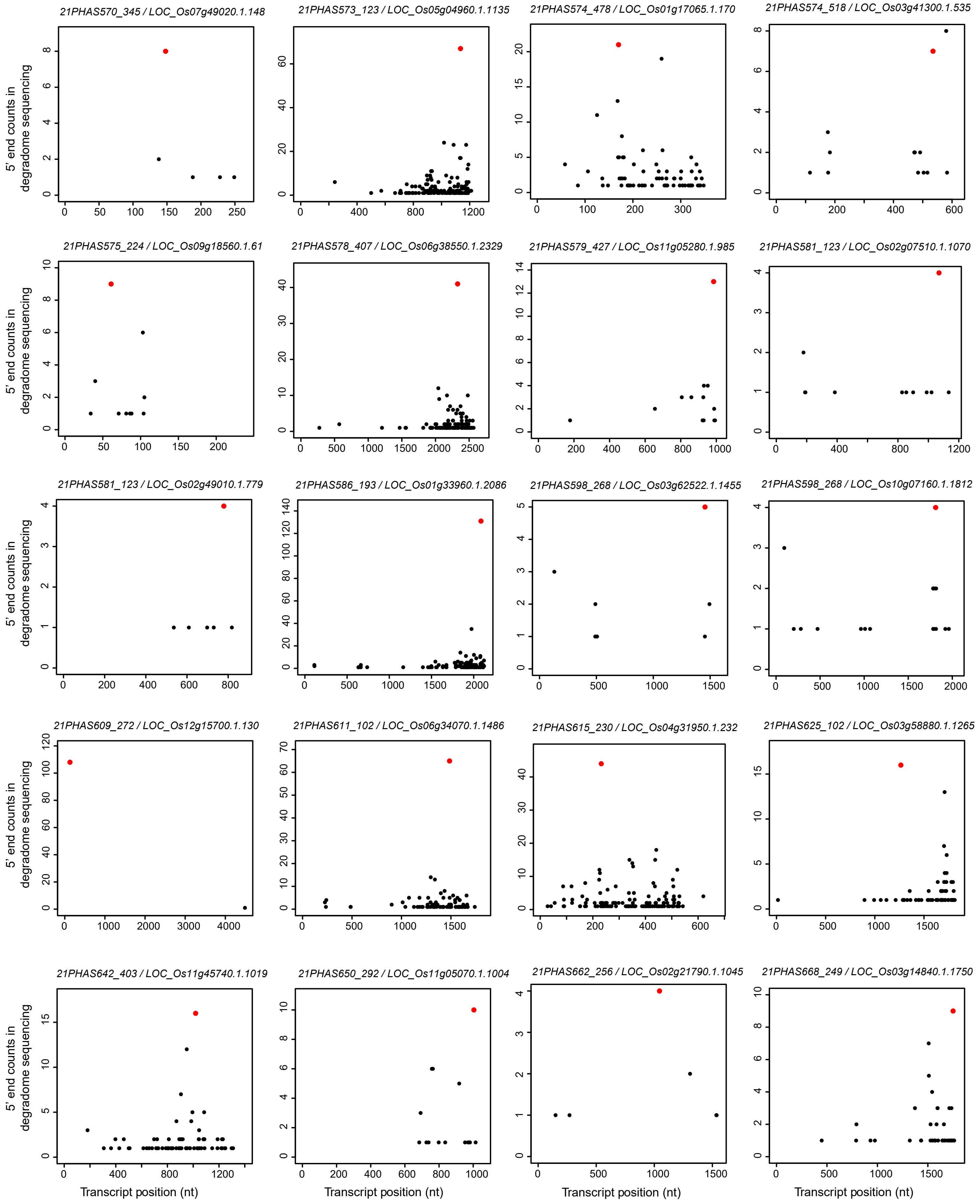
21PHAS570_345 / LOC_Os07g49020.1.148
21PHAS573_123 / LOC_Os05g04960.1.1135
21PHAS574_478 / LOC_Os01g17065.1.170
21PHAS574_518 / LOC_Os03g41300.1.535
21PHAS575_224 / LOC_Os09g18560.1.61
21PHAS578_407 / LOC_Os06g38550.1.2329
21PHAS579_427 / LOC_Os11g05280.1.985
21PHAS581_123 / LOC_Os02g07510.1.1070
21PHAS581_123 / LOC_Os02g49010.1.779
21PHAS586_193 / LOC_Os01g33960.1.2086
21PHAS598_268 / LOC_Os03g62522.1.1455
21PHAS598_268 / LOC_Os10g07160.1.1812
21PHAS609_272 / LOC_Os12g15700.1.130
21PHAS611_102 / LOC_Os06g34070.1.1486
21PHAS615_230 / LOC_Os04g31950.1.232
21PHAS625_102 / LOC_Os03g58880.1.1265
21PHAS642_403 / LOC_Os11g45740.1.1019
21PHAS650_292 / LOC_Os11g05070.1.1004
21PHAS662_256 / LOC_Os02g21790.1.1045
21PHAS668_249 / LOC_Os03g14840.1.1750
5' end counts in degradome sequencing
Transcript position (nt)

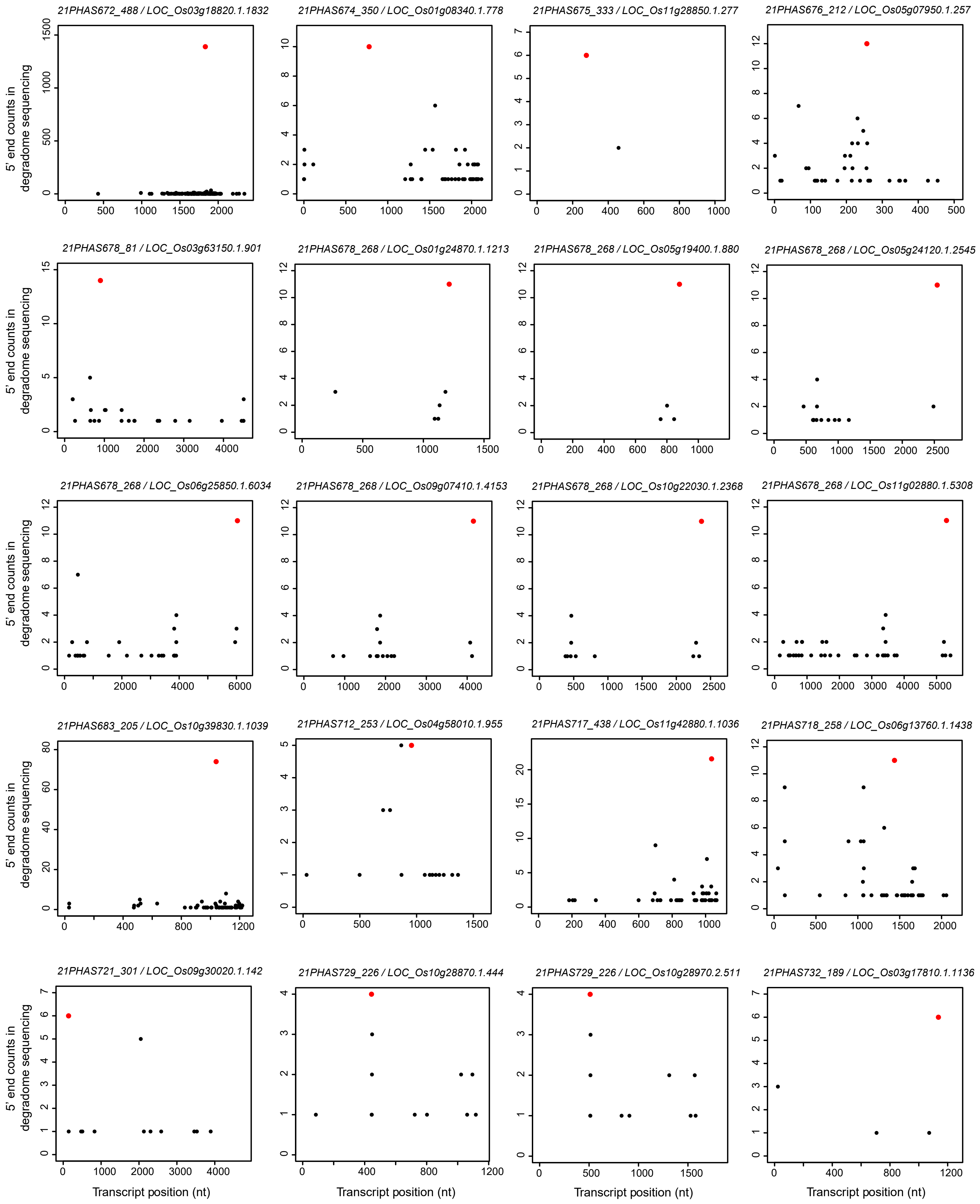
21PHAS672_488 / LOC_Os03g18820.1.1832
21PHAS674_350 / LOC_Os01g08340.1.778
21PHAS675_333 / LOC_Os11g28850.1.277
21PHAS676_212 / LOC_Os05g07950.1.257
21PHAS678_81 / LOC_Os03g63150.1.901
21PHAS678_268 / LOC_Os01g24870.1.1213
21PHAS678_268 / LOC_Os05g19400.1.880
21PHAS678_268 / LOC_Os05g24120.1.2545
21PHAS678_268 / LOC_Os06g25850.1.6034
21PHAS678_268 / LOC_Os09g07410.1.4153
21PHAS678_268 / LOC_Os10g22030.1.2368
21PHAS678_268 / LOC_Os11g02880.1.5308
21PHAS683_205 / LOC_Os10g39830.1.1039
21PHAS712_253 / LOC_Os04g58010.1.955
21PHAS717_438 / LOC_Os11g42880.1.1036
21PHAS718_258 / LOC_Os06g13760.1.1438
21PHAS721_301 / LOC_Os09g30020.1.142
21PHAS729_226 / LOC_Os10g28870.1.444
21PHAS729_226 / LOC_Os10g28970.2.511
21PHAS732_189 / LOC_Os03g17810.1.1136
5' end counts in degradome sequencing
Transcript position (nt)

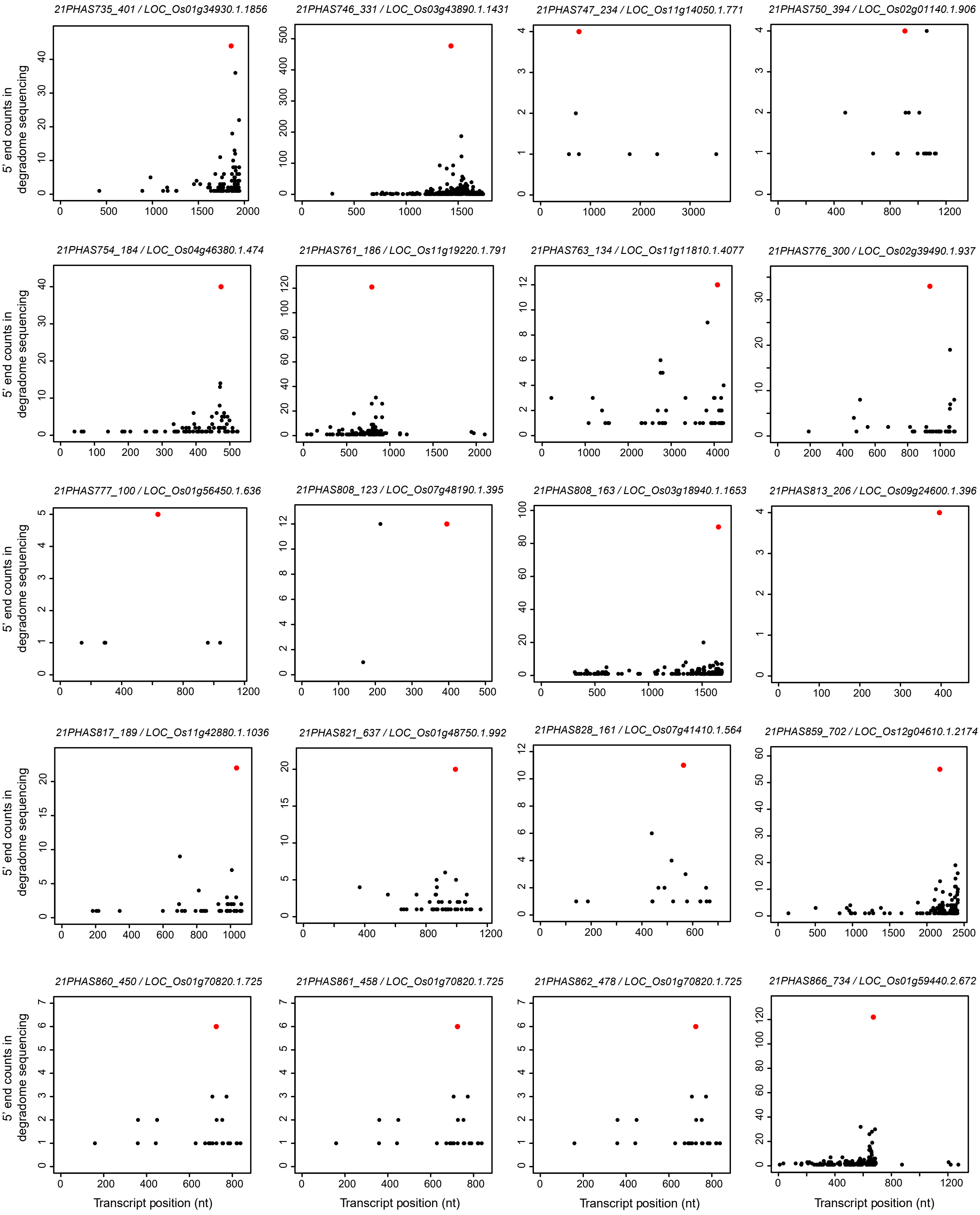
21PHAS735_401 / LOC_Os01g34930.1.1856
21PHAS746_331 / LOC_Os03g43890.1.1431
21PHAS747_234 / LOC_Os11g14050.1.771
21PHAS750_394 / LOC_Os02g01140.1.906
21PHAS754_184 / LOC_Os04g46380.1.474
21PHAS761_186 / LOC_Os11g19220.1.791
21PHAS763_134 / LOC_Os11g11810.1.4077
21PHAS776_300 / LOC_Os02g39490.1.937
21PHAS777_100 / LOC_Os01g56450.1.636
21PHAS808_123 / LOC_Os07g48190.1.395
21PHAS808_163 / LOC_Os03g18940.1.1653
21PHAS813_206 / LOC_Os09g24600.1.396
21PHAS817_189 / LOC_Os11g42880.1.1036
21PHAS821_637 / LOC_Os01g48750.1.992
21PHAS828_161 / LOC_Os07g41410.1.564
21PHAS859_702 / LOC_Os12g04610.1.2174
21PHAS860_450 / LOC_Os01g70820.1.725
21PHAS861_458 / LOC_Os01g70820.1.725
21PHAS862_478 / LOC_Os01g70820.1.725
21PHAS866_734 / LOC_Os01g59440.2.672
5' end counts in degradome sequencing
Transcript position (nt)

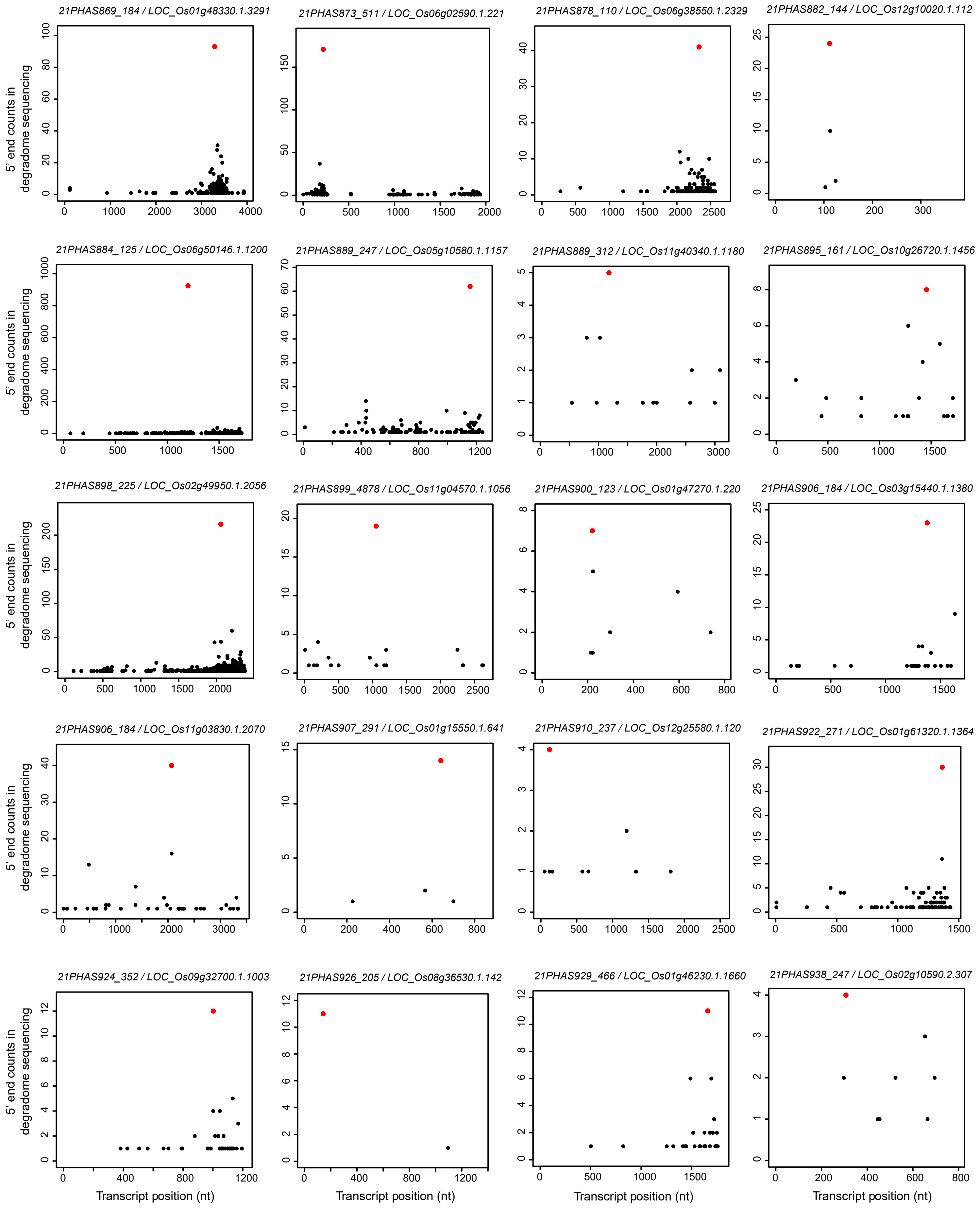
21PHAS869_184 / LOC_Os01g48330.1.3291
21PHAS873_511 / LOC_Os06g02590.1.221
21PHAS878_110 / LOC_Os06g38550.1.2329
21PHAS882_144 / LOC_Os12g10020.1.112
21PHAS884_125 / LOC_Os06g50146.1.1200
21PHAS889_247 / LOC_Os05g10580.1.1157
21PHAS889_312 / LOC_Os11g40340.1.1180
21PHAS895_161 / LOC_Os10g26720.1.1456
21PHAS898_225 / LOC_Os02g49950.1.2056
21PHAS899_4878 / LOC_Os11g04570.1.1056
21PHAS900_123 / LOC_Os01g47270.1.220
21PHAS906_184 / LOC_Os03g15440.1.1380
21PHAS906_184 / LOC_Os11g03830.1.2070
21PHAS907_291 / LOC_Os01g15550.1.641
21PHAS910_237 / LOC_Os12g25580.1.120
21PHAS922_271 / LOC_Os01g61320.1.1364
21PHAS924_352 / LOC_Os09g32700.1.1003
21PHAS926_205 / LOC_Os08g36530.1.142
21PHAS929_466 / LOC_Os01g46230.1.1660
21PHAS938_247 / LOC_Os02g10590.2.307
5' end counts in degradome sequencing
Transcript position (nt)

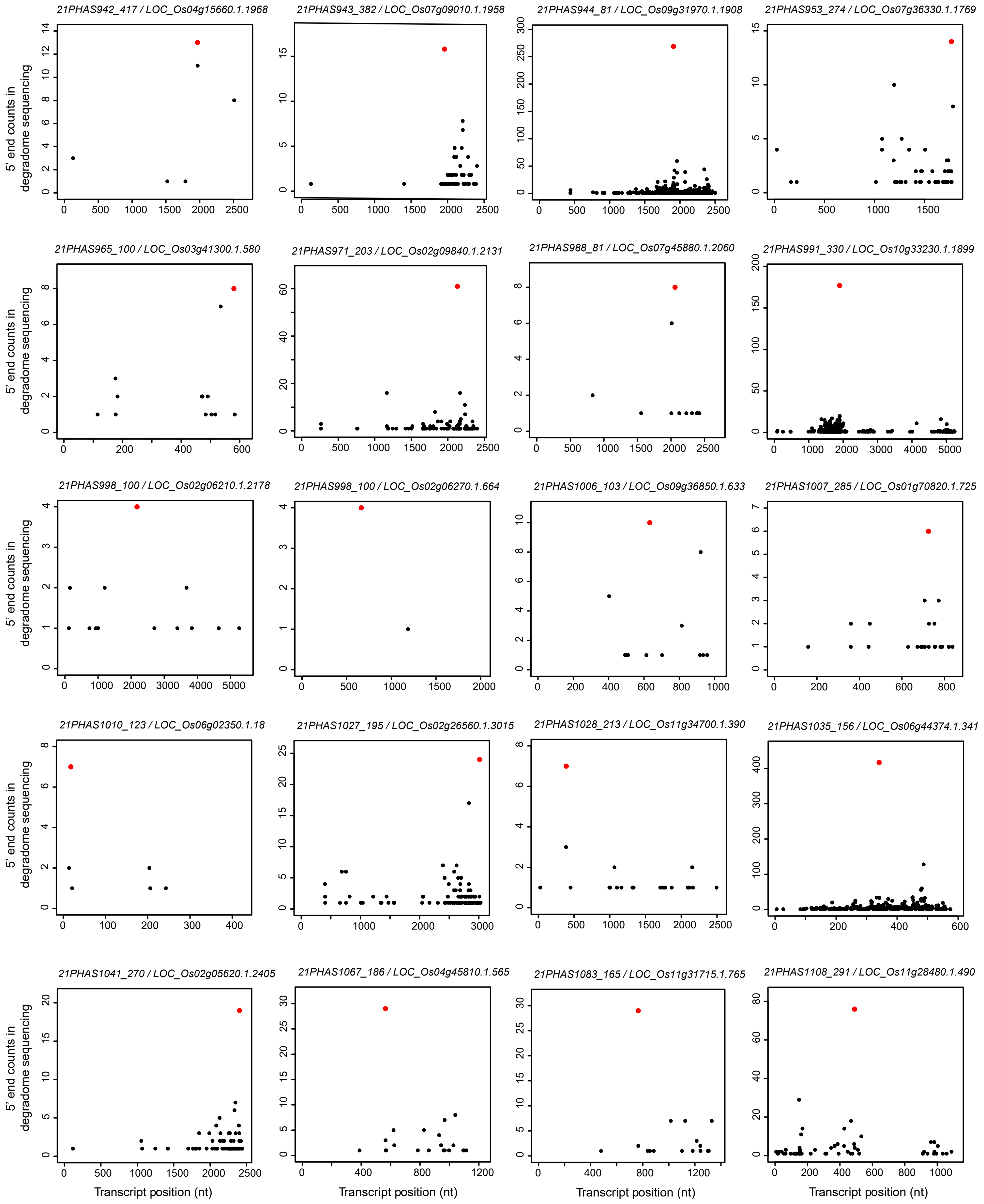
21PHAS942_417 / LOC_Os04g15660.1.1968
21PHAS943_382 / LOC_Os07g09010.1.1958
21PHAS944_81 / LOC_Os09g31970.1.1908
21PHAS953_274 / LOC_Os07g36330.1.1769
21PHAS965_100 / LOC_Os03g41300.1.580
21PHAS971_203 / LOC_Os02g09840.1.2131
21PHAS988_81 / LOC_Os07g45880.1.2060
21PHAS991_330 / LOC_Os10g33230.1.1899
21PHAS998_100 / LOC_Os02g06210.1.2178
21PHAS998_100 / LOC_Os02g06270.1.664
21PHAS1006_103 / LOC_Os09g36850.1.633
21PHAS1007_285 / LOC_Os01g70820.1.725
21PHAS1010_123 / LOC_Os06g02350.1.18
21PHAS1027_195 / LOC_Os02g26560.1.3015
21PHAS1028_213 / LOC_Os11g34700.1.390
21PHAS1035_156 / LOC_Os06g44374.1.341
21PHAS1041_270 / LOC_Os02g05620.1.2405
21PHAS1067_186 / LOC_Os04g45810.1.565
21PHAS1083_165 / LOC_Os11g31715.1.765
21PHAS1108_291 / LOC_Os11g28480.1.490
5' end counts in degradome sequencing
Transcript position (nt)

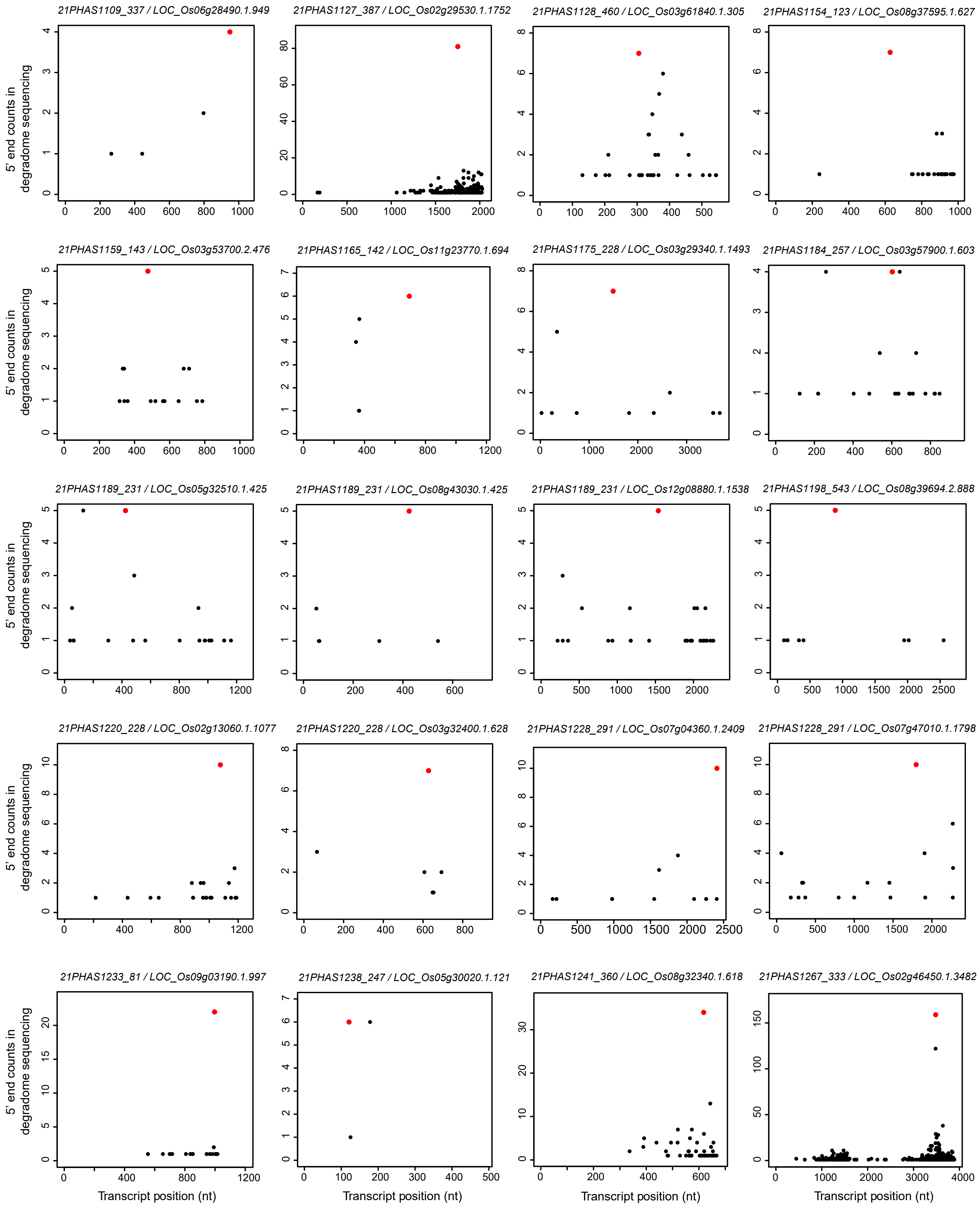
21PHAS1109_337 / LOC_Os06g28490.1.949
21PHAS1127_387 / LOC_Os02g29530.1.1752
21PHAS1128_460 / LOC_Os03g61840.1.305
21PHAS1154_123 / LOC_Os08g37595.1.627
21PHAS1159_143 / LOC_Os03g53700.2.476
21PHAS1165_142 / LOC_Os11g23770.1.694
21PHAS1175_228 / LOC_Os03g29340.1.1493
21PHAS1184_257 / LOC_Os03g57900.1.603
21PHAS1189_231 / LOC_Os05g32510.1.425
21PHAS1189_231 / LOC_Os08g43030.1.425
21PHAS1189_231 / LOC_Os12g08880.1.1538
21PHAS1198_543 / LOC_Os08g39694.2.888
21PHAS1220_228 / LOC_Os02g13060.1.1077
21PHAS1220_228 / LOC_Os03g32400.1.628
21PHAS1228_291 / LOC_Os07g04360.1.2409
21PHAS1228_291 / LOC_Os07g47010.1.1798
21PHAS1233_81 / LOC_Os09g03190.1.997
21PHAS1238_247 / LOC_Os05g30020.1.121
21PHAS1241_360 / LOC_Os08g32340.1.618
21PHAS1267_333 / LOC_Os02g46450.1.3482
5' end counts in degradome sequencing
Transcript position (nt)

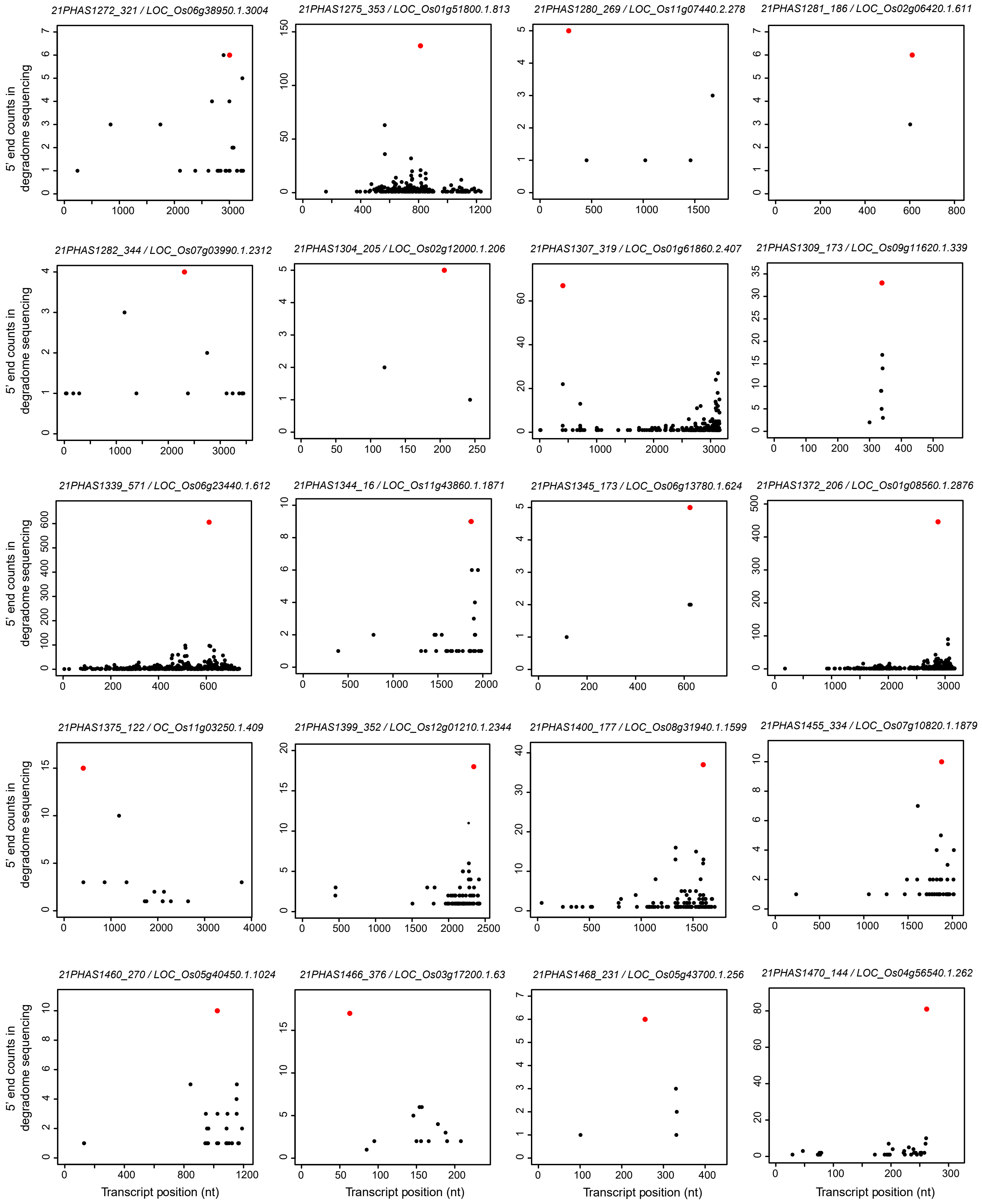

21PHAS1272_321 / LOC_Os06g38950.1.3004
21PHAS1275_353 / LOC_Os01g51800.1.813
21PHAS1280_269 / LOC_Os11g07440.2.278
21PHAS1281_186 / LOC_Os02g06420.1.611
21PHAS1282_344 / LOC_Os07g03990.1.2312
21PHAS1304_205 / LOC_Os02g12000.1.206
21PHAS1307_319 / LOC_Os01g61860.2.407
21PHAS1309_173 / LOC_Os09g11620.1.339
21PHAS1339_571 / LOC_Os06g23440.1.612
21PHAS1344_16 / LOC_Os11g43860.1.1871
21PHAS1345_173 / LOC_Os06g13780.1.624
21PHAS1372_206 / LOC_Os01g08560.1.2876
21PHAS1375_122 / OC_Os11g03250.1.409
21PHAS1399_352 / LOC_Os12g01210.1.2344
21PHAS1400_177 / LOC_Os08g31940.1.1599
21PHAS1455_334 / LOC_Os07g10820.1.1879
21PHAS1460_270 / LOC_Os05g40450.1.1024
21PHAS1466_376 / LOC_Os03g17200.1.63
21PHAS1468_231 / LOC_Os05g43700.1.256
21PHAS1470_144 / LOC_Os04g56540.1.262
5' end counts in degradome sequencing
Transcript position (nt)

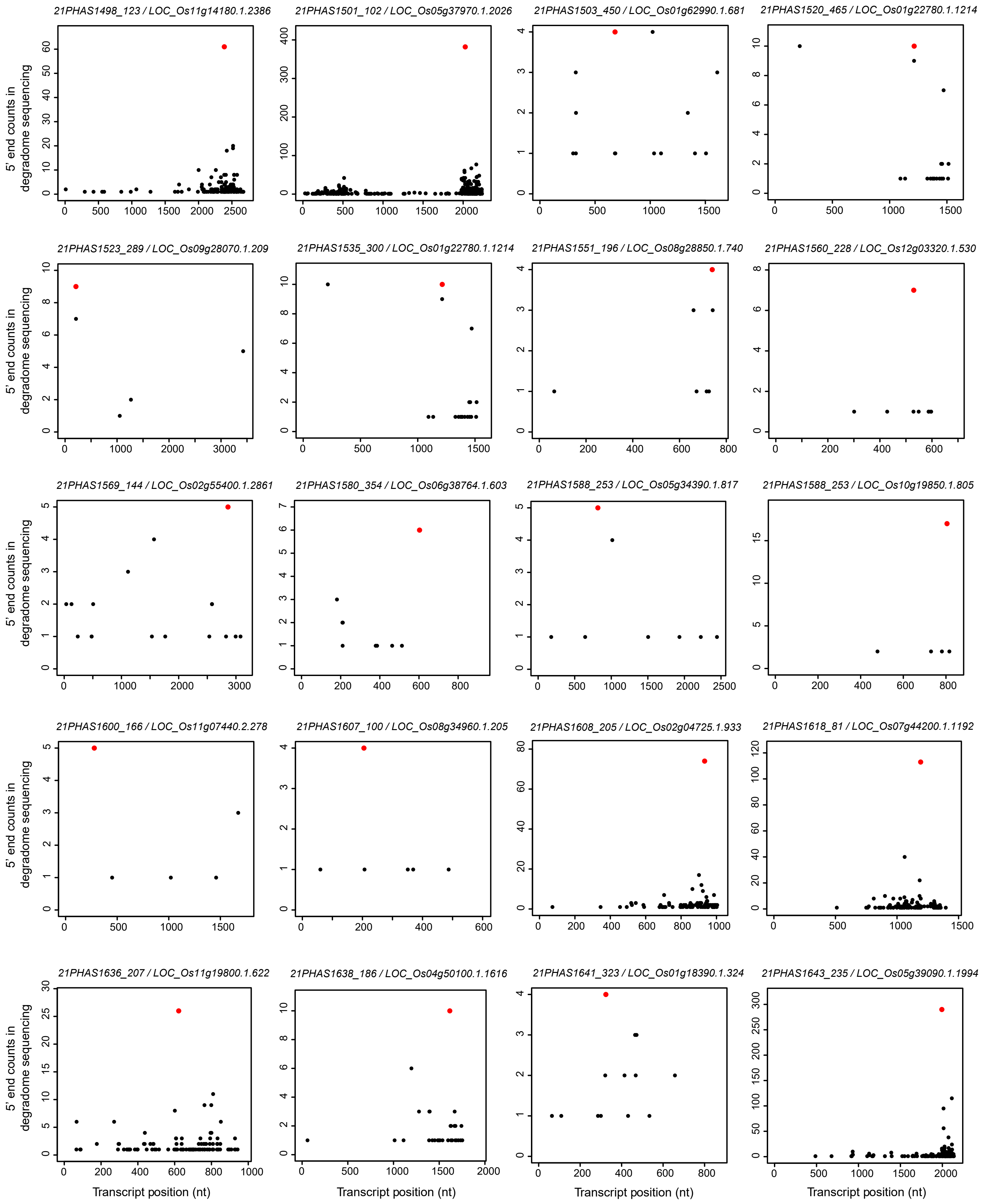
21PHAS1498_123 / LOC_Os11g14180.1.2386
21PHAS1501_102 / LOC_Os05g37970.1.2026
21PHAS1503_450 / LOC_Os01g62990.1.681
21PHAS1520_465 / LOC_Os01g22780.1.1214
21PHAS1523_289 / LOC_Os09g28070.1.209
21PHAS1535_300 / LOC_Os01g22780.1.1214
21PHAS1551_196 / LOC_Os08g28850.1.740
21PHAS1560_228 / LOC_Os12g03320.1.530
21PHAS1569_144 / LOC_Os02g55400.1.2861
21PHAS1580_354 / LOC_Os06g38764.1.603
21PHAS1588_253 / LOC_Os05g34390.1.817
21PHAS1588_253 / LOC_Os10g19850.1.805
21PHAS1600_166 / LOC_Os11g07440.2.278
21PHAS1607_100 / LOC_Os08g34960.1.205
21PHAS1608_205 / LOC_Os02g04725.1.933
21PHAS1618_81 / LOC_Os07g44200.1.1192
21PHAS1636_207 / LOC_Os11g19800.1.622
21PHAS1638_186 / LOC_Os04g50100.1.1616
21PHAS1641_323 / LOC_Os01g18390.1.324
21PHAS1643_235 / LOC_Os05g39090.1.1994
5' end counts in degradome sequencing
Transcript position (nt)

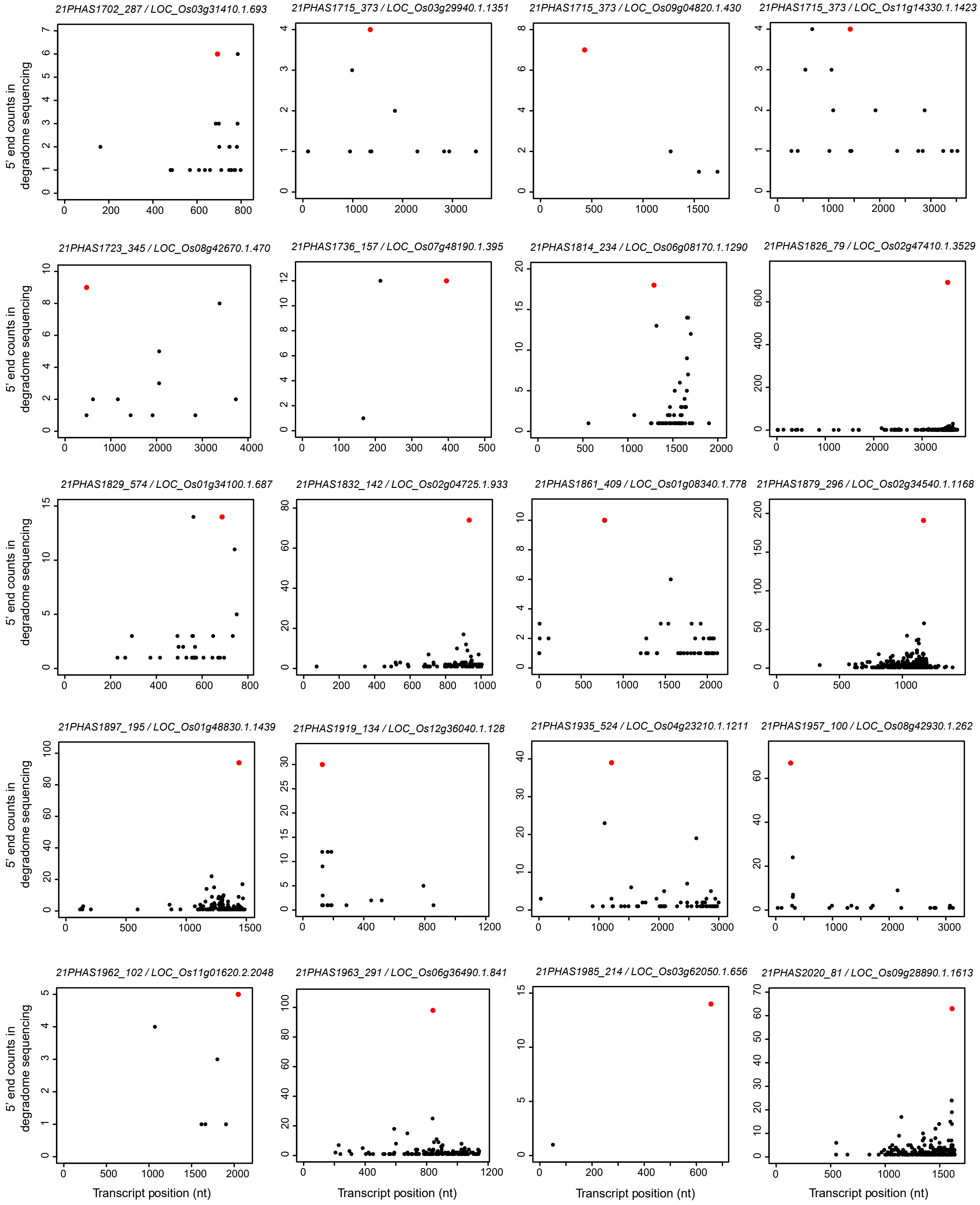
21PHAS1702_287 / LOC_Os03g31410.1.693
21PHAS1715_373 / LOC_Os03g29940.1.1351
21PHAS1715_373 / LOC_Os09g04820.1.430
21PHAS1715_373 / LOC_Os11g14330.1.1423
21PHAS1723_345 / LOC_Os08g42670.1.470
21PHAS1736_157 / LOC_Os07g48190.1.395
21PHAS1814_234 / LOC_Os06g08170.1.1290
21PHAS1826_79 / LOC_Os02g47410.1.3529
21PHAS1829_574 / LOC_Os01g34100.1.687
21PHAS1832_142 / LOC_Os02g04725.1.933
21PHAS1861_409 / LOC_Os01g08340.1.778
21PHAS1879_296 / LOC_Os02g34540.1.1168
21PHAS1897_195 / LOC_Os01g48830.1.1439
21PHAS1919_134 / LOC_Os12g36040.1.128
21PHAS1935_524 / LOC_Os04g23210.1.1211
21PHAS1957_100 / LOC_Os08g42930.1.262
21PHAS1962_102 / LOC_Os11g01620.2.2048
21PHAS1963_291 / LOC_Os06g36490.1.841
21PHAS1985_214 / LOC_Os03g62050.1.656
21PHAS2020_81 / LOC_Os09g28890.1.1613
5' end counts in degradome sequencing
Transcript position (nt)

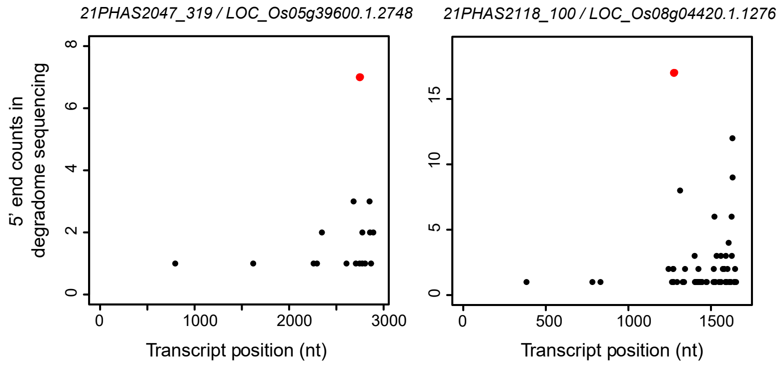

Supplementary Fig. 6 Validation of 21-nt phasiRNA targets in early prohase I meiocytes by degradome sequencing. Plots showing the distribution of the degradome tags along 21-nt phasiRNA targets. The x-axis represents the nucleotide position of each target mRNA (5'-3'). The y-axis represents the relative frequency of degradome tags. The red dot represents degradometag at +1 position of 21-nt phasiRNA-guided cleavage site.

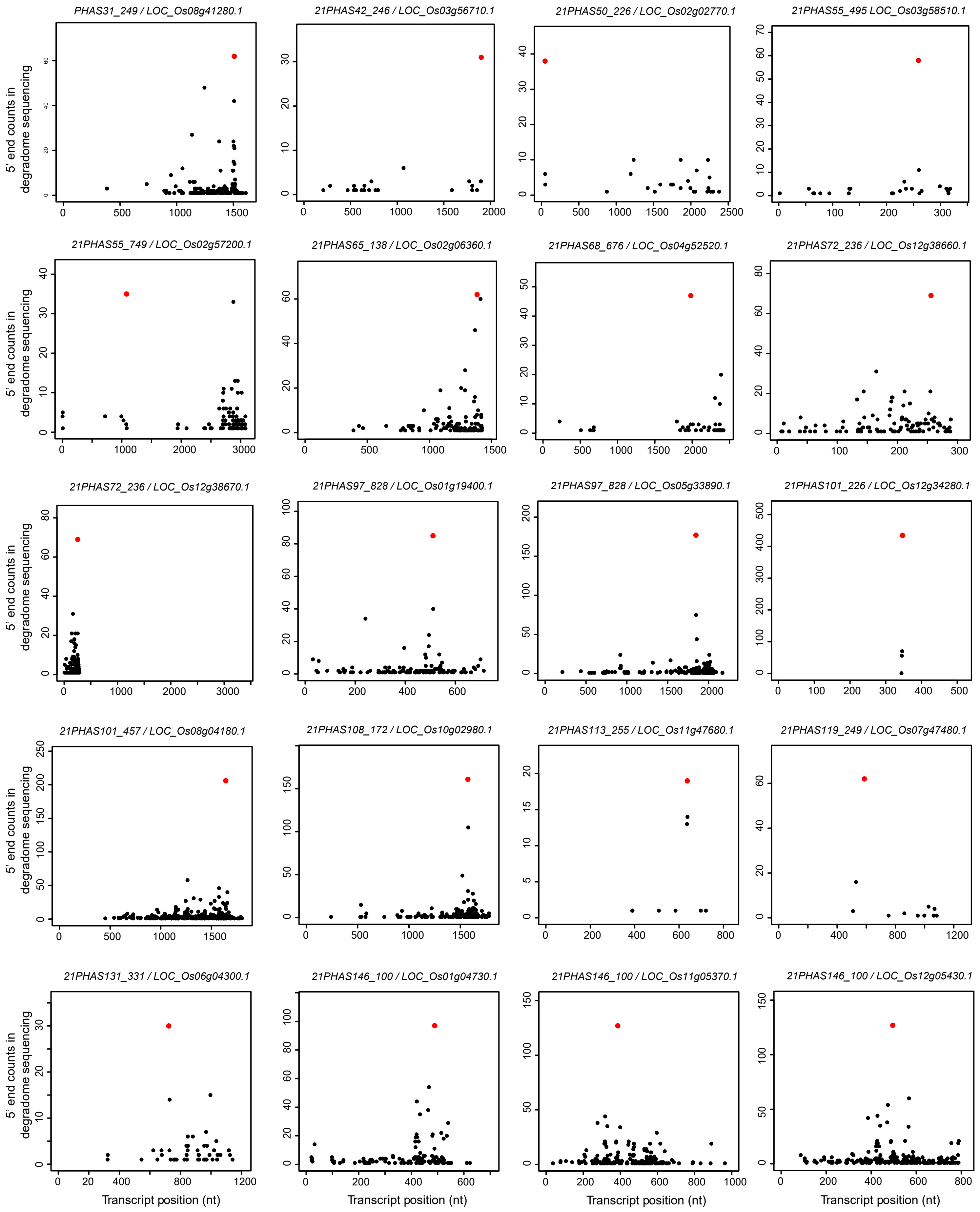
PHAS31_249 / LOC_Os08g41280.1
21PHAS42_246 / LOC_Os03g56710.1
21PHAS50_226 / LOC_Os02g02770.1
21PHAS55_495 LOC_Os03g58510.1
21PHAS55_749 / LOC_Os02g57200.1
21PHAS65_138 / LOC_Os02g06360.1
21PHAS68_676 / LOC_Os04g52520.1
21PHAS72_236 / LOC_Os12g38660.1
21PHAS72_236 / LOC_Os12g38670.1
21PHAS97_828 / LOC_Os01g19400.1
21PHAS97_828 / LOC_Os05g33890.1
21PHAS101_226 / LOC_Os12g34280.1
21PHAS101_457 / LOC_Os08g04180.1
21PHAS108_172 / LOC_Os10g02980.1
21PHAS113_255 / LOC_Os11g47680.1
21PHAS119_249 / LOC_Os07g47480.1
21PHAS131_331 / LOC_Os06g04300.1
21PHAS146_100 / LOC_Os01g04730.1
21PHAS146_100 / LOC_Os11g05370.1
21PHAS146_100 / LOC_Os12g05430.1
5' end counts in degradome sequencing
Transcript position (nt)

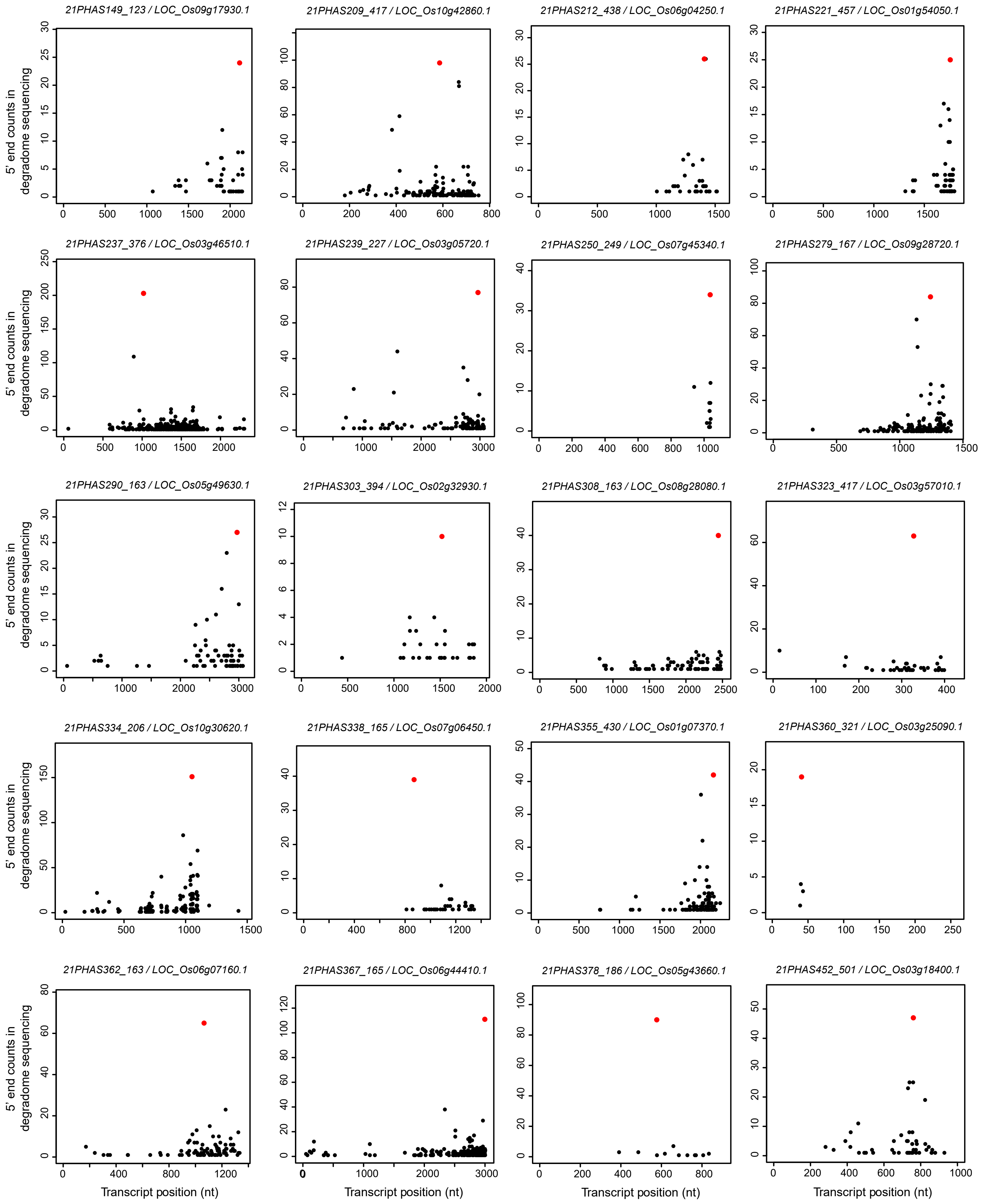
21PHAS149_123 / LOC_Os09g17930.1
21PHAS209_417 / LOC_Os10g42860.1
21PHAS212_438 / LOC_Os06g04250.1
21PHAS221_457 / LOC_Os01g54050.1
21PHAS237_376 / LOC_Os03g46510.1
21PHAS239_227 / LOC_Os03g05720.1
21PHAS250_249 / LOC_Os07g45340.1
21PHAS279_167 / LOC_Os09g28720.1
21PHAS290_163 / LOC_Os05g49630.1
21PHAS303_394 / LOC_Os02g32930.1
21PHAS308_163 / LOC_Os08g28080.1
21PHAS323_417 / LOC_Os03g57010.1
21PHAS334_206 / LOC_Os10g30620.1
21PHAS338_165 / LOC_Os07g06450.1
21PHAS355_430 / LOC_Os01g07370.1
21PHAS360_321 / LOC_Os03g25090.1
21PHAS362_163 / LOC_Os06g07160.1
21PHAS367_165 / LOC_Os06g44410.1
21PHAS378_186 / LOC_Os05g43660.1
21PHAS452_501 / LOC_Os03g18400.1
5' end counts in degradome sequencing
Transcript position (nt)

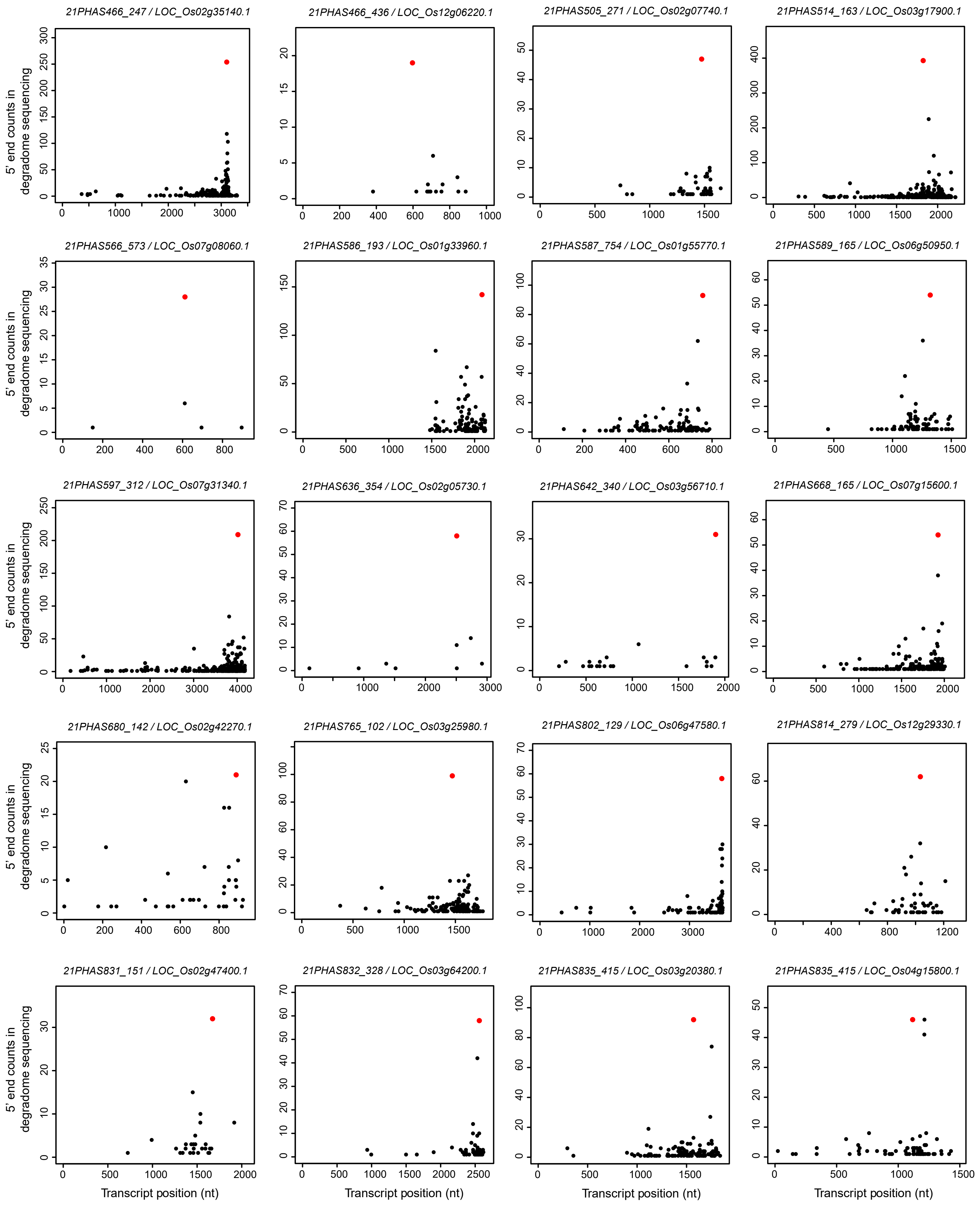
21PHAS466_247 / LOC_Os02g35140.1
21PHAS466_436 / LOC_Os12g06220.1
21PHAS505_271 / LOC_Os02g07740.1
21PHAS514_163 / LOC_Os03g17900.1
21PHAS566_573 / LOC_Os07g08060.1
21PHAS586_193 / LOC_Os01g33960.1
21PHAS587_754 / LOC_Os01g55770.1
21PHAS589_165 / LOC_Os06g50950.1
21PHAS597_312 / LOC_Os07g31340.1
21PHAS636_354 / LOC_Os02g05730.1
21PHAS642_340 / LOC_Os03g56710.1
21PHAS668_165 / LOC_Os07g15600.1
21PHAS680_142 / LOC_Os02g42270.1
21PHAS765_102 / LOC_Os03g25980.1
21PHAS802_129 / LOC_Os06g47580.1
21PHAS814_279 / LOC_Os12g29330.1
21PHAS831_151 / LOC_Os02g47400.1
21PHAS832_328 / LOC_Os03g64200.1
21PHAS835_415 / LOC_Os03g20380.1
21PHAS835_415 / LOC_Os04g15800.1
5' end counts in degradome sequencing
Transcript position (nt)

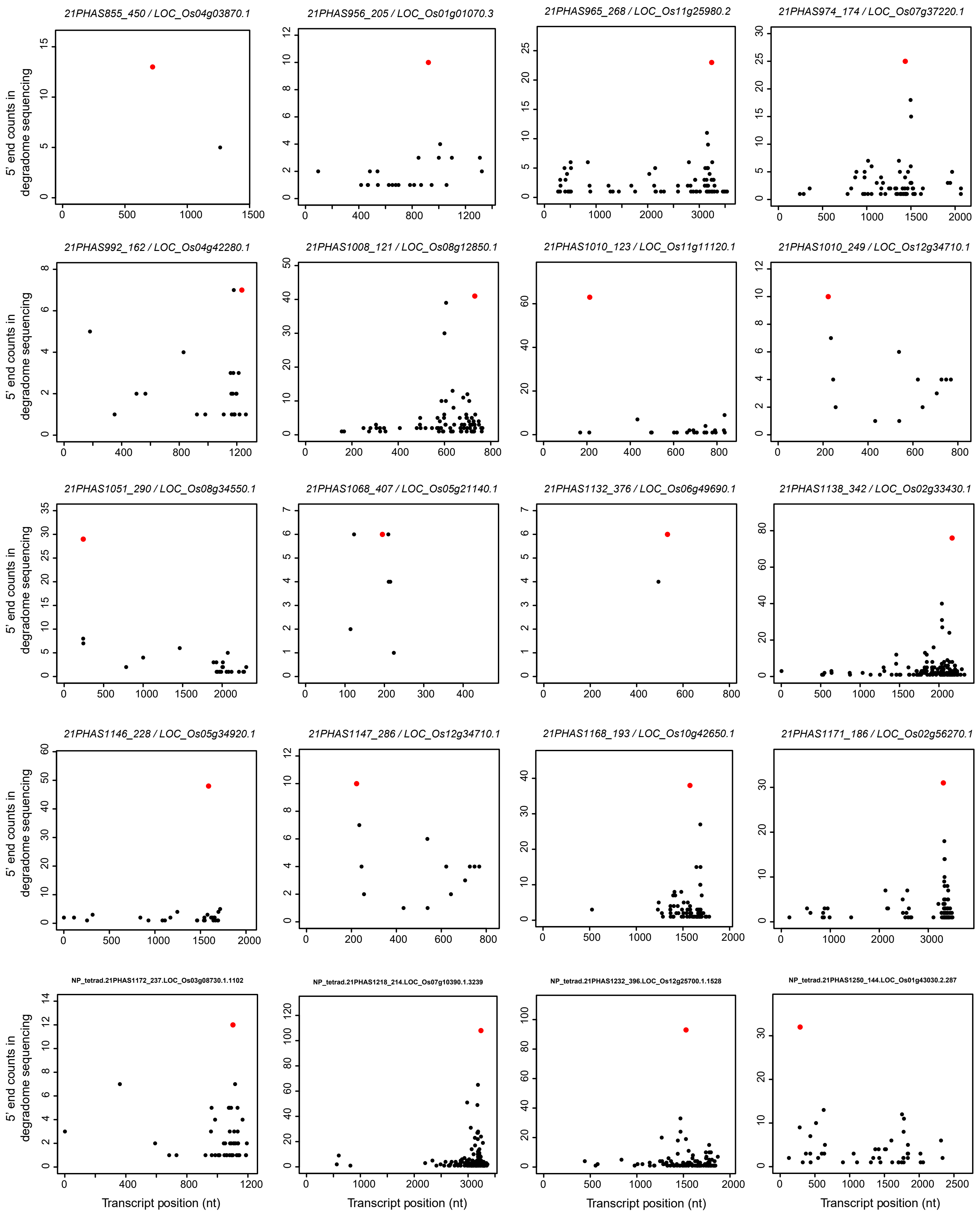
21PHAS855_450 / LOC_Os04g03870.1
21PHAS956_205 / LOC_Os01g01070.3
21PHAS965_268 / LOC_Os11g25980.2
21PHAS974_174 / LOC_Os07g37220.1
21PHAS992_162 / LOC_Os04g42280.1
21PHAS1008_121 / LOC_Os08g12850.1
21PHAS1010_123 / LOC_Os11g11120.1
21PHAS1010_249 / LOC_Os12g34710.1
21PHAS1051_290 / LOC_Os08g34550.1
21PHAS1068_407 / LOC_Os05g21140.1
21PHAS1132_376 / LOC_Os06g49690.1
21PHAS1138_342 / LOC_Os02g33430.1
21PHAS1146_228 / LOC_Os05g34920.1
21PHAS1147_286 / LOC_Os12g34710.1
21PHAS1168_193 / LOC_Os10g42650.1
21PHAS1171_186 / LOC_Os02g56270.1
NP_tetrad.21PHAS1172_237.LOC_Os03g08730.1.1102
NP_tetrad.21PHAS1218_214.LOC_Os07g10390.1.3239
NP_tetrad.21PHAS1232_396.LOC_Os12g25700.1.1528
NP_tetrad.21PHAS1250_144.LOC_Os01g43030.2.287
5' end counts in degradome sequencing
Transcript position (nt)

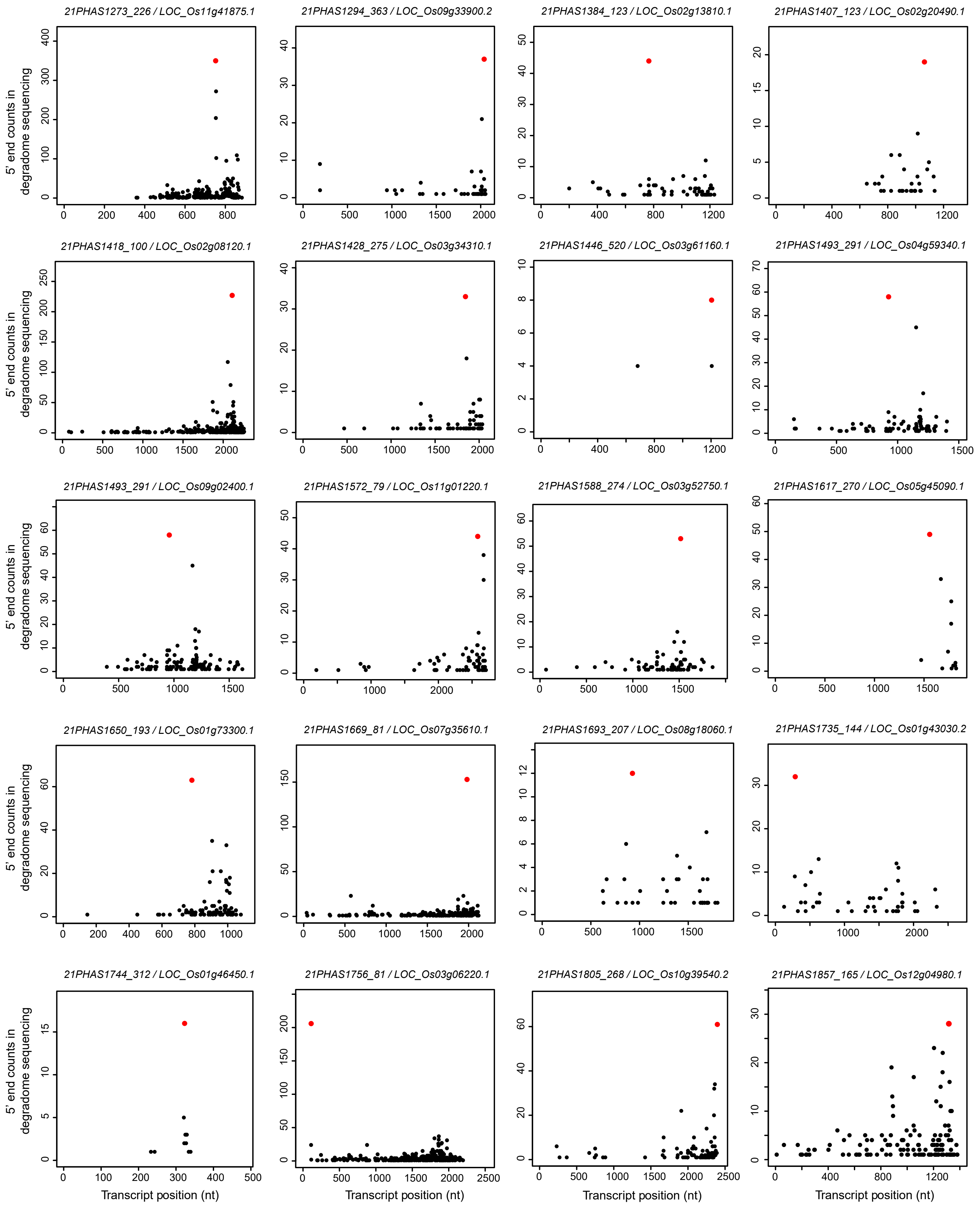
21PHAS1273_226 / LOC_Os11g41875.1
21PHAS1294_363 / LOC_Os09g33900.2
21PHAS1384_123 / LOC_Os02g13810.1
21PHAS1407_123 / LOC_Os02g20490.1
21PHAS1418_100 / LOC_Os02g08120.1
21PHAS1428_275 / LOC_Os03g34310.1
21PHAS1446_520 / LOC_Os03g61160.1
21PHAS1493_291 / LOC_Os04g59340.1
21PHAS1493_291 / LOC_Os09g02400.1
21PHAS1572_79 / LOC_Os11g01220.1
21PHAS1588_274 / LOC_Os03g52750.1
21PHAS1617_270 / LOC_Os05g45090.1
21PHAS1650_193 / LOC_Os01g73300.1
21PHAS1669_81 / LOC_Os07g35610.1
21PHAS1693_207 / LOC_Os08g18060.1
21PHAS1735_144 / LOC_Os01g43030.2
21PHAS1744_312 / LOC_Os01g46450.1
21PHAS1756_81 / LOC_Os03g06220.1
21PHAS1805_268 / LOC_Os10g39540.2
21PHAS1857_165 / LOC_Os12g04980.1
5' end counts in degradome sequencing
Transcript position (nt)

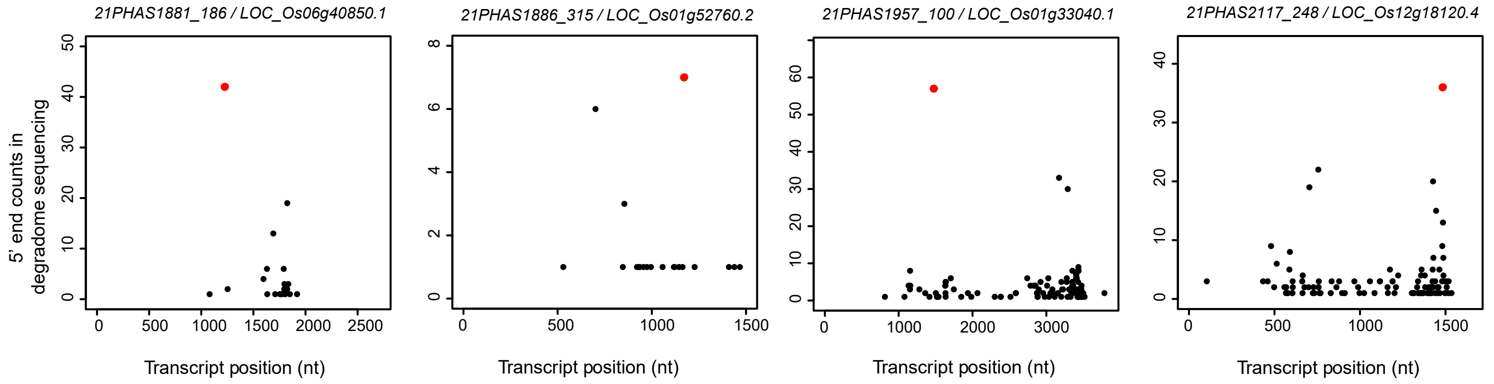

Supplementary Fig. 7 Validation of 21-nt phasiRNA targets in tetrads by degradome sequencing. Plots showing the distribution of the degradome tags along 21-nt phasiRNA targets. The x-axis represents the nucleotide position of each target mRNA (5'-3'). The y-axis represents the relative frequency of degradome tags. The red dot represents degradome tag at +1 position of 21-nt phasiRNA-guided cleavage site.

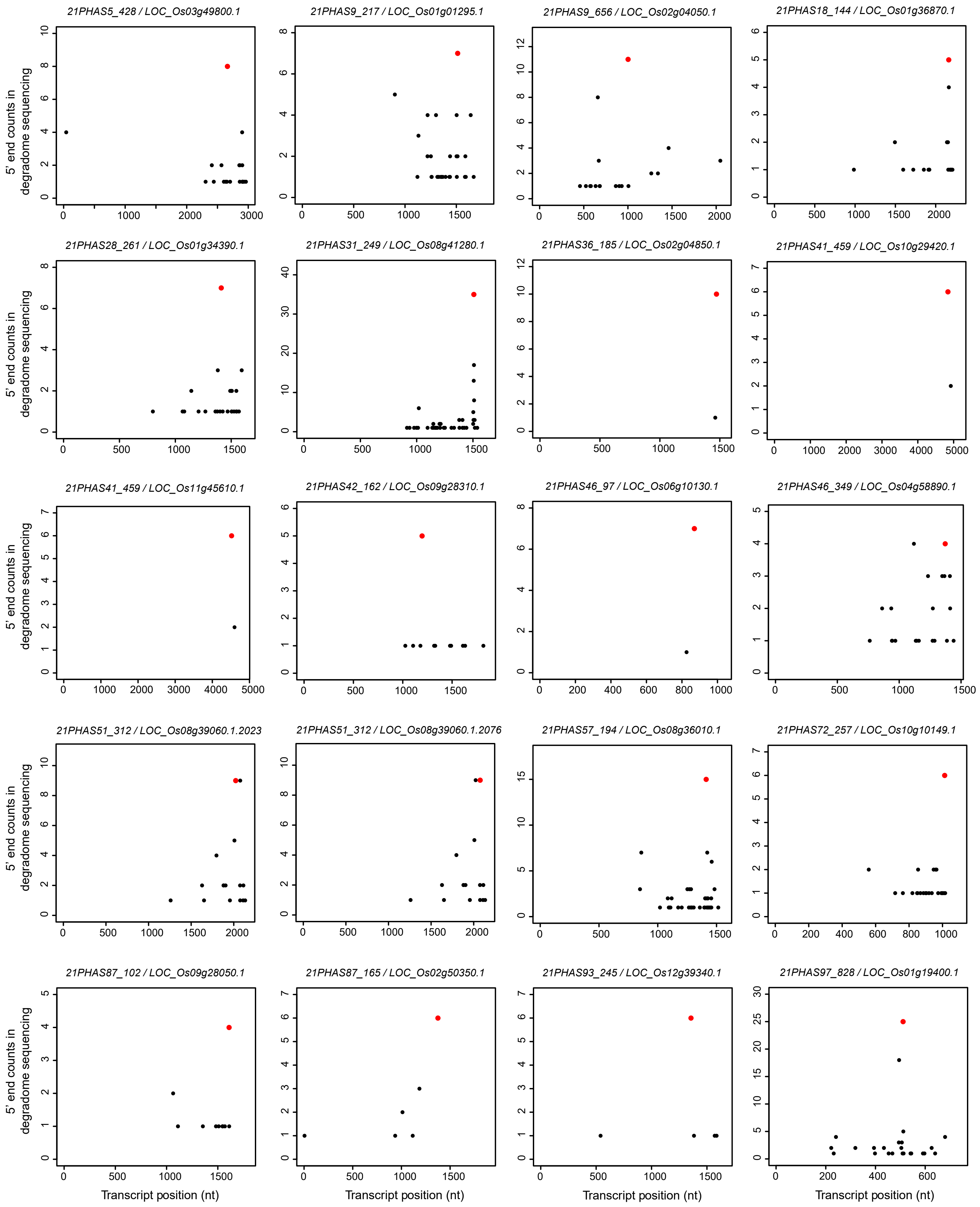
21PHAS5_428 / LOC_Os03g49800.1
21PHAS9_217 / LOC_Os01g01295.1
21PHAS9_656 / LOC_Os02g04050.1
21PHAS18_144 / LOC_Os01g36870.1
21PHAS28_261 / LOC_Os01g34390.1
21PHAS31_249 / LOC_Os08g41280.1
21PHAS36_185 / LOC_Os02g04850.1
21PHAS41_459 / LOC_Os10g29420.1
21PHAS41_459 / LOC_Os11g45610.1
21PHAS42_162 / LOC_Os09g28310.1
21PHAS46_97 / LOC_Os06g10130.1
21PHAS46_349 / LOC_Os04g58890.1
21PHAS51_312 / LOC_Os08g39060.1.2023
21PHAS51_312 / LOC_Os08g39060.1.2076
21PHAS57_194 / LOC_Os08g36010.1
21PHAS72_257 / LOC_Os10g10149.1
21PHAS87_102 / LOC_Os09g28050.1
21PHAS87_165 / LOC_Os02g50350.1
21PHAS93_245 / LOC_Os12g39340.1
21PHAS97_828 / LOC_Os01g19400.1
5' end counts in degradome sequencing
Transcript position (nt)

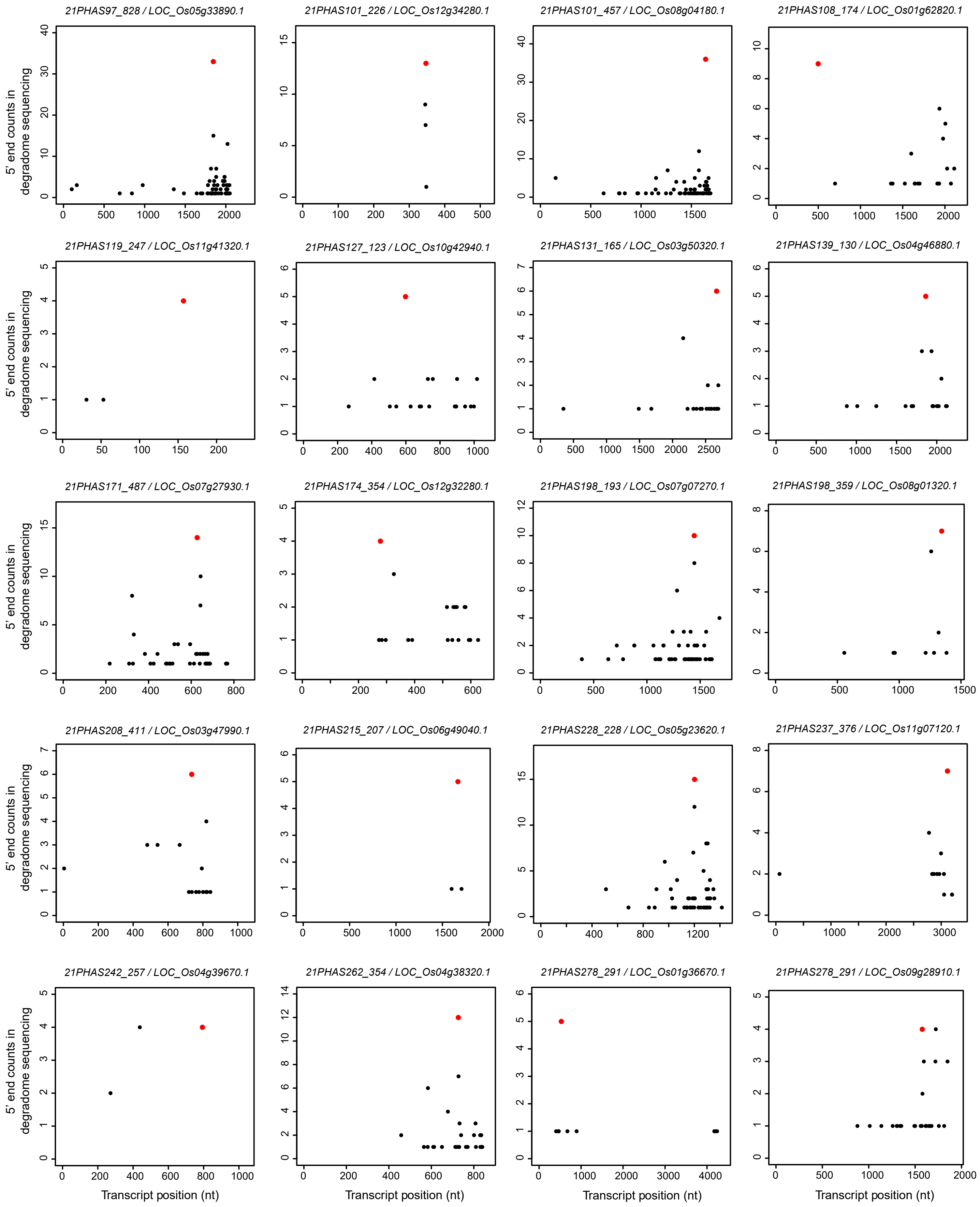

21PHAS97_828 / LOC_Os05g33890.1
21PHAS101_226 / LOC_Os12g34280.1
21PHAS101_457 / LOC_Os08g04180.1
21PHAS108_174 / LOC_Os01g62820.1
21PHAS119_247 / LOC_Os11g41320.1
21PHAS127_123 / LOC_Os10g42940.1
21PHAS131_165 / LOC_Os03g50320.1
21PHAS139_130 / LOC_Os04g46880.1
21PHAS171_487 / LOC_Os07g27930.1
21PHAS174_354 / LOC_Os12g32280.1
21PHAS198_193 / LOC_Os07g07270.1
21PHAS198_359 / LOC_Os08g01320.1
21PHAS208_411 / LOC_Os03g47990.1
21PHAS215_207 / LOC_Os06g49040.1
21PHAS228_228 / LOC_Os05g23620.1
21PHAS237_376 / LOC_Os11g07120.1
21PHAS242_257 / LOC_Os04g39670.1
21PHAS262_354 / LOC_Os04g38320.1
21PHAS278_291 / LOC_Os01g36670.1
21PHAS278_291 / LOC_Os09g28910.1
5' end counts in degradome sequencing
Transcript position (nt)

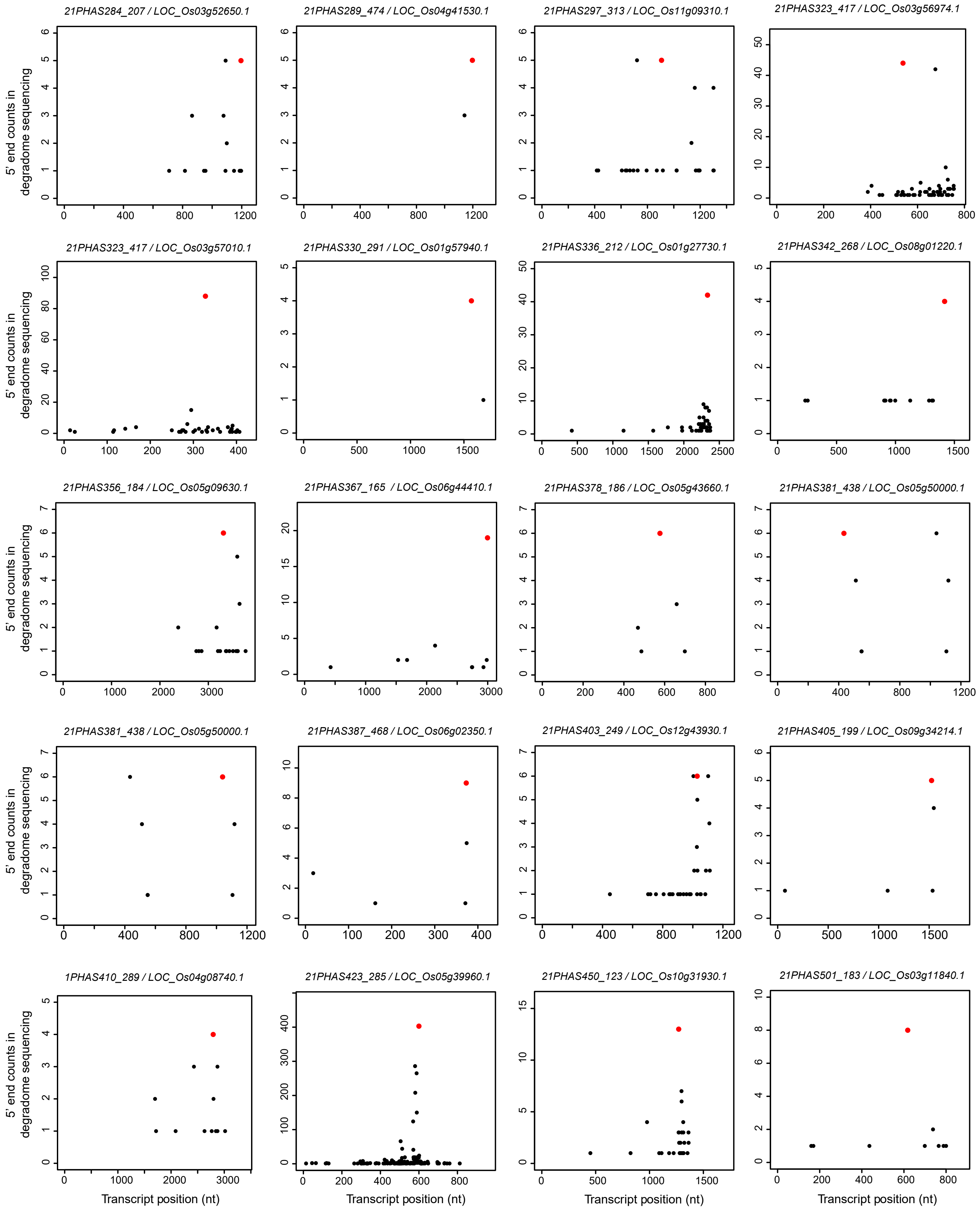

21PHAS284_207 / LOC_Os03g52650.1
21PHAS289_474 / LOC_Os04g41530.1
21PHAS297_313 / LOC_Os11g09310.1
21PHAS323_417 / LOC_Os03g56974.1
21PHAS323_417 / LOC_Os03g57010.1
21PHAS330_291 / LOC_Os01g57940.1
21PHAS336_212 / LOC_Os01g27730.1
21PHAS342_268 / LOC_Os08g01220.1
21PHAS356_184 / LOC_Os05g09630.1
21PHAS367_165 / LOC_Os06g44410.1
21PHAS378_186 / LOC_Os05g43660.1
21PHAS381_438 / LOC_Os05g50000.1
21PHAS381_438 / LOC_Os05g50000.1
21PHAS387_468 / LOC_Os06g02350.1
21PHAS403_249 / LOC_Os12g43930.1
21PHAS405_199 / LOC_Os09g34214.1
1PHAS410_289 / LOC_Os04g08740.1
21PHAS423_285 / LOC_Os05g39960.1
21PHAS450_123 / LOC_Os10g31930.1
21PHAS501_183 / LOC_Os03g11840.1
5' end counts in degradome sequencing
Transcript position (nt)

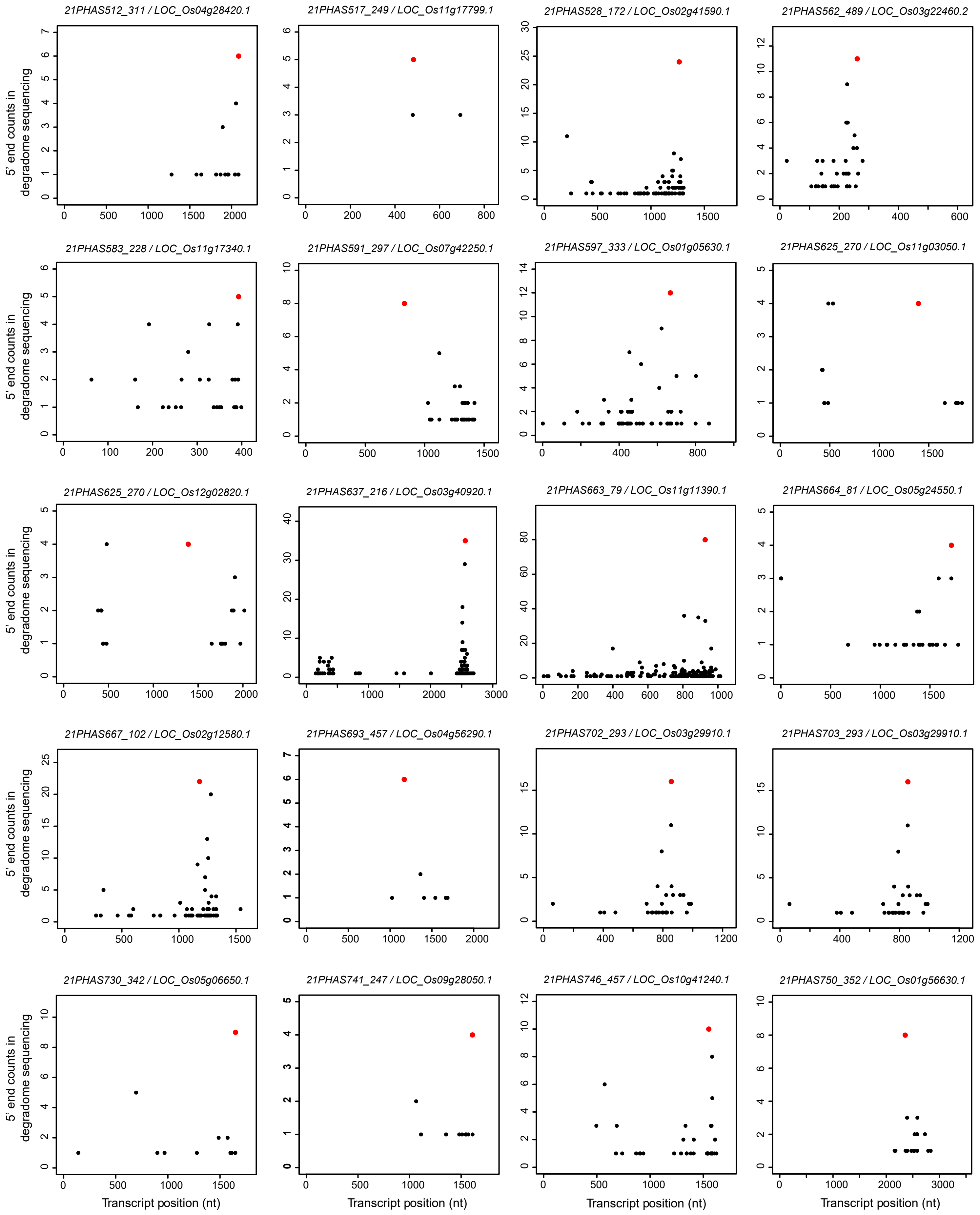

21PHAS512_311 / LOC_Os04g28420.1
21PHAS517_249 / LOC_Os11g17799.1
21PHAS528_172 / LOC_Os02g41590.1
21PHAS562_489 / LOC_Os03g22460.2
21PHAS583_228 / LOC_Os11g17340.1
21PHAS591_297 / LOC_Os07g42250.1
21PHAS597_333 / LOC_Os01g05630.1
21PHAS625_270 / LOC_Os11g03050.1
21PHAS625_270 / LOC_Os12g02820.1
21PHAS637_216 / LOC_Os03g40920.1
21PHAS663_79 / LOC_Os11g11390.1
21PHAS664_81 / LOC_Os05g24550.1
21PHAS667_102 / LOC_Os02g12580.1
21PHAS693_457 / LOC_Os04g56290.1
21PHAS702_293 / LOC_Os03g29910.1
21PHAS703_293 / LOC_Os03g29910.1
21PHAS730_342 / LOC_Os05g06650.1
21PHAS741_247 / LOC_Os09g28050.1
21PHAS746_457 / LOC_Os10g41240.1
21PHAS750_352 / LOC_Os01g56630.1
5' end counts in degradome sequencing
Transcript position (nt)

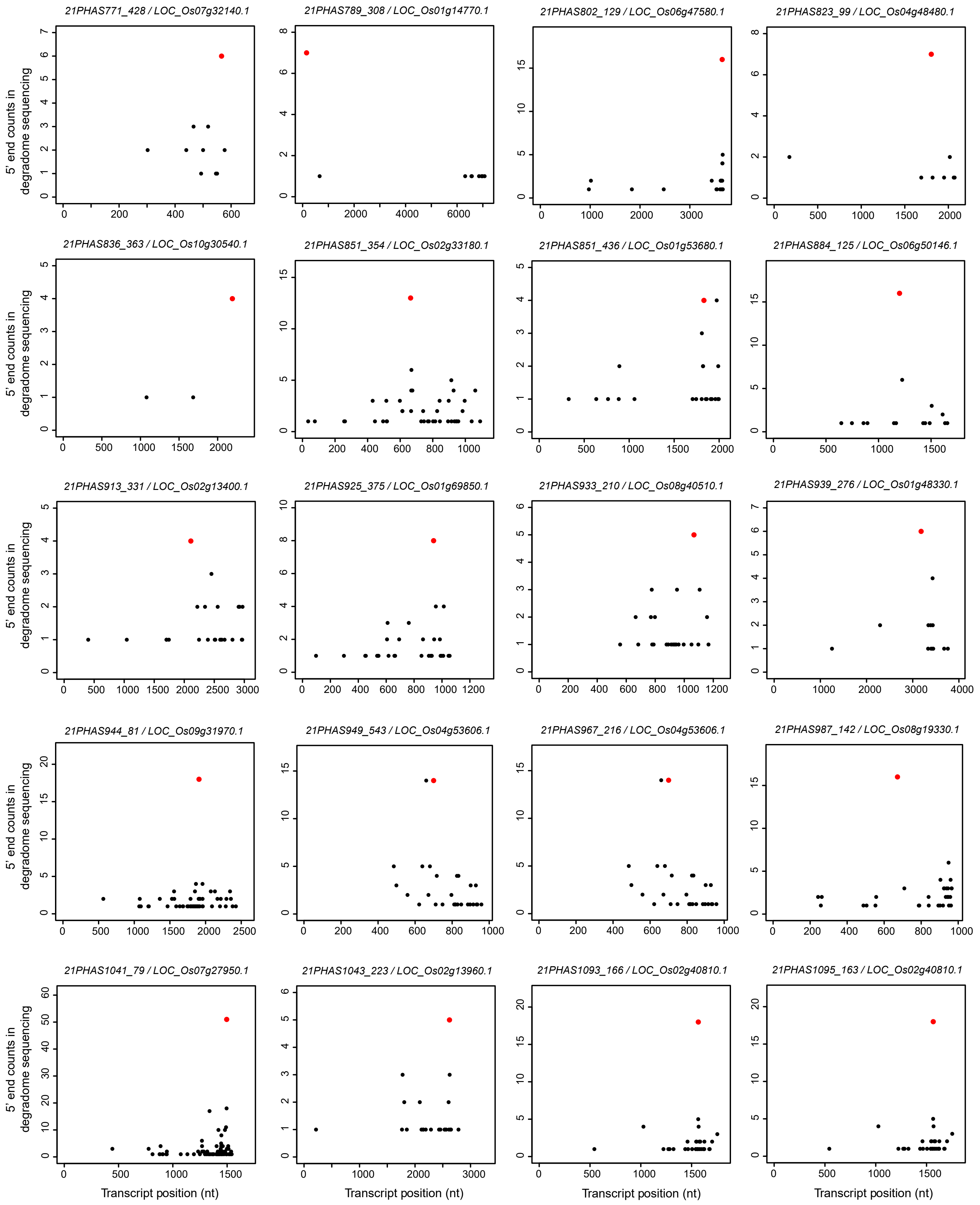

21PHAS771_428 / LOC_Os07g32140.1
21PHAS789_308 / LOC_Os01g14770.1
21PHAS802_129 / LOC_Os06g47580.1
21PHAS823_99 / LOC_Os04g48480.1
21PHAS836_363 / LOC_Os10g30540.1
21PHAS851_354 / LOC_Os02g33180.1
21PHAS851_436 / LOC_Os01g53680.1
21PHAS884_125 / LOC_Os06g50146.1
21PHAS913_331 / LOC_Os02g13400.1
21PHAS925_375 / LOC_Os01g69850.1
21PHAS933_210 / LOC_Os08g40510.1
21PHAS939_276 / LOC_Os01g48330.1
21PHAS944_81 / LOC_Os09g31970.1
21PHAS949_543 / LOC_Os04g53606.1
21PHAS967_216 / LOC_Os04g53606.1
21PHAS987_142 / LOC_Os08g19330.1
21PHAS1041_79 / LOC_Os07g27950.1
21PHAS1043_223 / LOC_Os02g13960.1
21PHAS1093_166 / LOC_Os02g40810.1
21PHAS1095_163 / LOC_Os02g40810.1
5' end counts in degradome sequencing
Transcript position (nt)

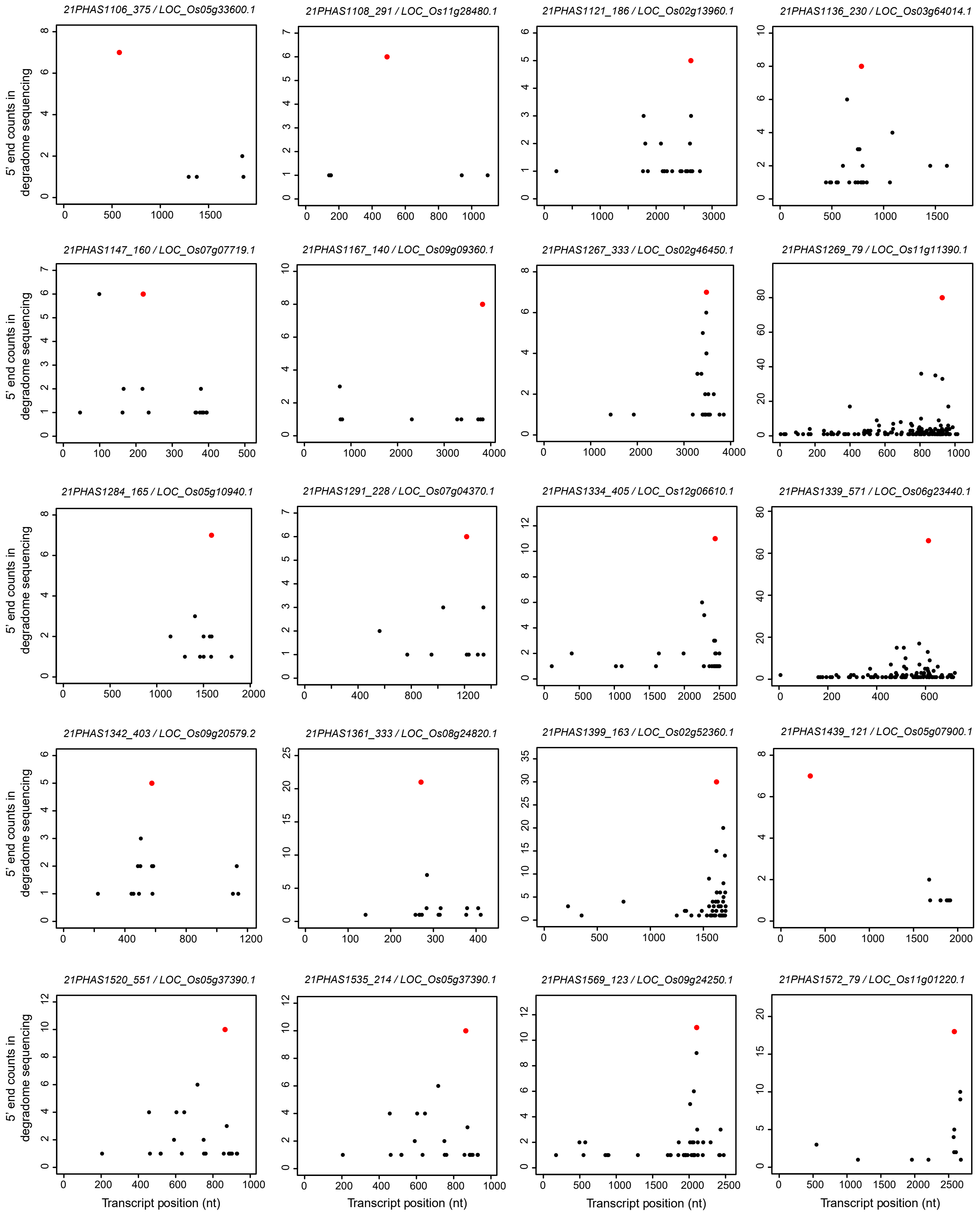
21PHAS1106_375 / LOC_Os05g33600.1
21PHAS1108_291 / LOC_Os11g28480.1
21PHAS1121_186 / LOC_Os02g13960.1
21PHAS1136_230 / LOC_Os03g64014.1
21PHAS1147_160 / LOC_Os07g07719.1
21PHAS1167_140 / LOC_Os09g09360.1
21PHAS1267_333 / LOC_Os02g46450.1
21PHAS1269_79 / LOC_Os11g11390.1
21PHAS1284_165 / LOC_Os05g10940.1
21PHAS1291_228 / LOC_Os07g04370.1
21PHAS1334_405 / LOC_Os12g06610.1
21PHAS1339_571 / LOC_Os06g23440.1
21PHAS1342_403 / LOC_Os09g20579.2
21PHAS1361_333 / LOC_Os08g24820.1
21PHAS1399_163 / LOC_Os02g52360.1
21PHAS1439_121 / LOC_Os05g07900.1
21PHAS1520_551 / LOC_Os05g37390.1
21PHAS1535_214 / LOC_Os05g37390.1
21PHAS1569_123 / LOC_Os09g24250.1
21PHAS1572_79 / LOC_Os11g01220.1
5' end counts in degradome sequencing
Transcript position (nt)

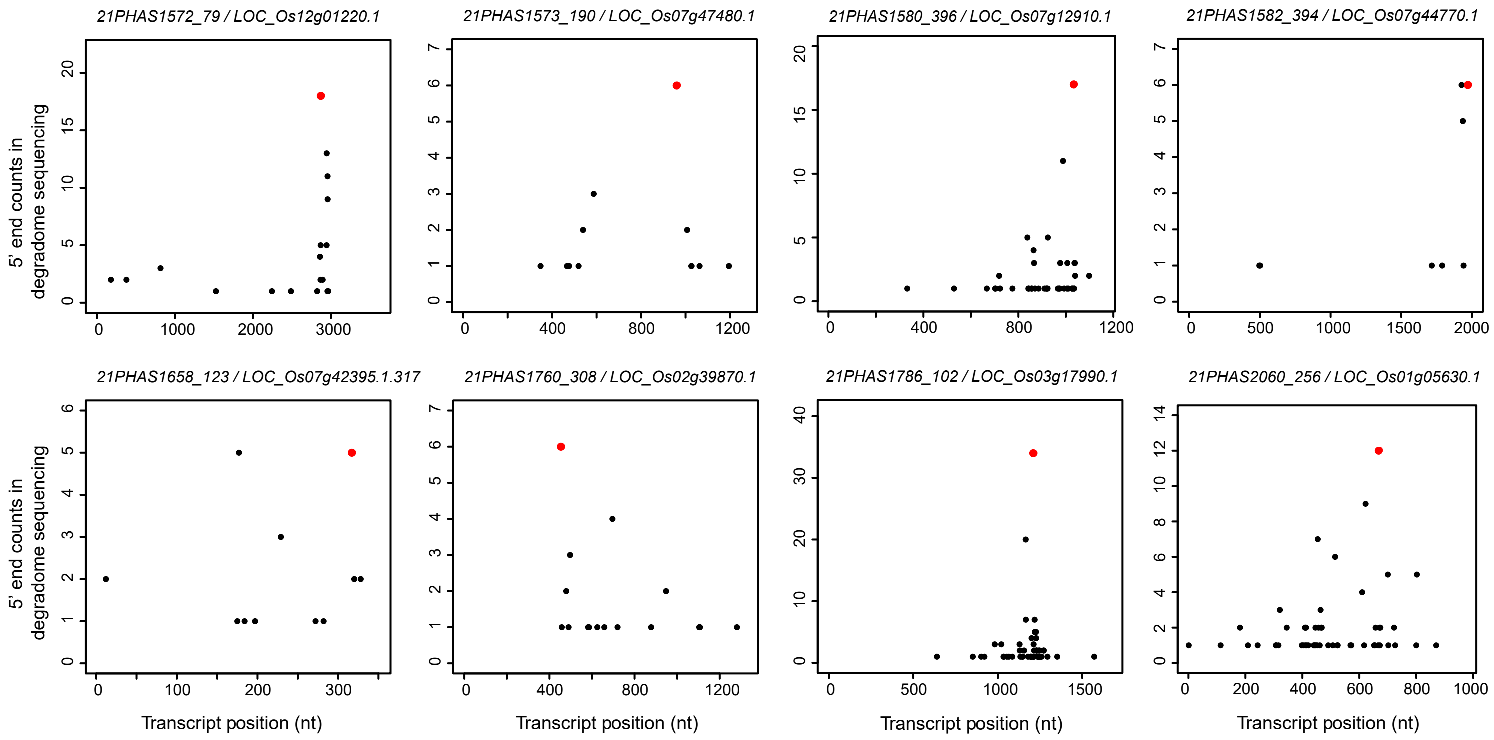

Supplementary Fig. 8 Validation of 21-nt phasiRNA targets in microspores by degradome sequencing. Plots showing the distribution of the degradome tags along 21-nt phasiRNA targets. The x-axis represents the nucleotide position of each target mRNA (5'-3'). The y-axis represents the relative frequency of degradome tags. The red dot represents degradome tag at +1 position of 21-nt phasiRNA-guided cleavage site.

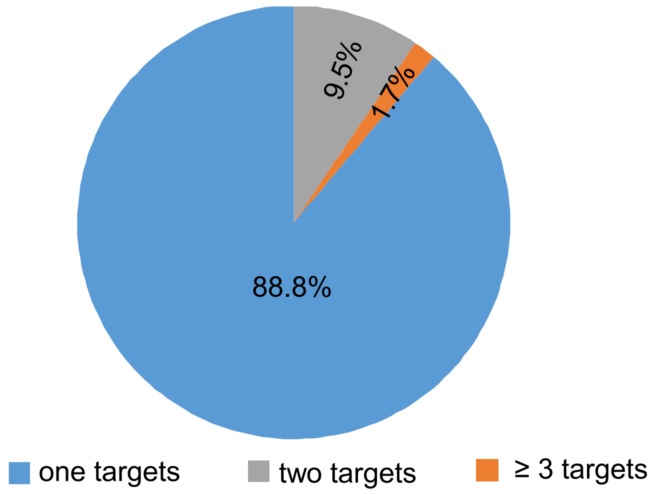

Supplementary Fig. 9. Analysis of *PHAS* loci with targets. Pie chart showing the percentages of *PHAS* loci that produce phasiRNAs targeting one, two and three or more genes, respectively.

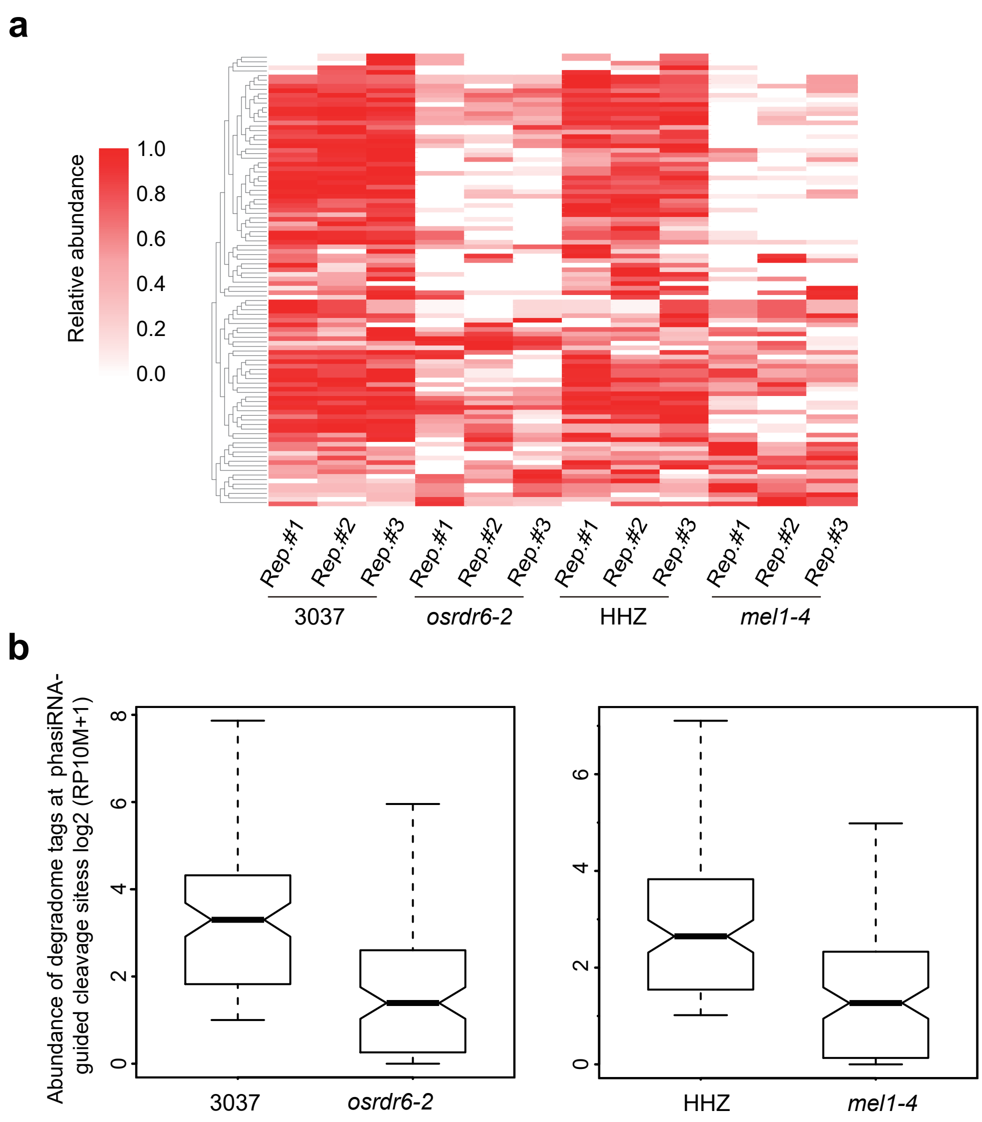

Supplementary Fig. 10. Relative abundances of degradome tags at phasiRNA-guided cleavage sites in 3037, *osrdr6-2*, HHZ and *mel1-4*. a, Heatmap showing the abundances of degradome tags at +1 positions of 21 -nt phasiRNA-guided cleavage sites in early prophase I meiocytes of *osrdr6-2*, *mel1-4* and their respective wild-type plants. Colors intensity represents the fractional density across the row of log2-transformed (RP10M+1) values. **b,** Box plots showing the log2-tranformed (RP10M+1) values in 3037, *osrdr6-2*, HHZ and *mel1-4*. The central line of the box represents the median while two bounds represent 25% quartile and 75% quartile, respectively. The whisker represents 1.5 × interquartile range of the lower or upper quartile.

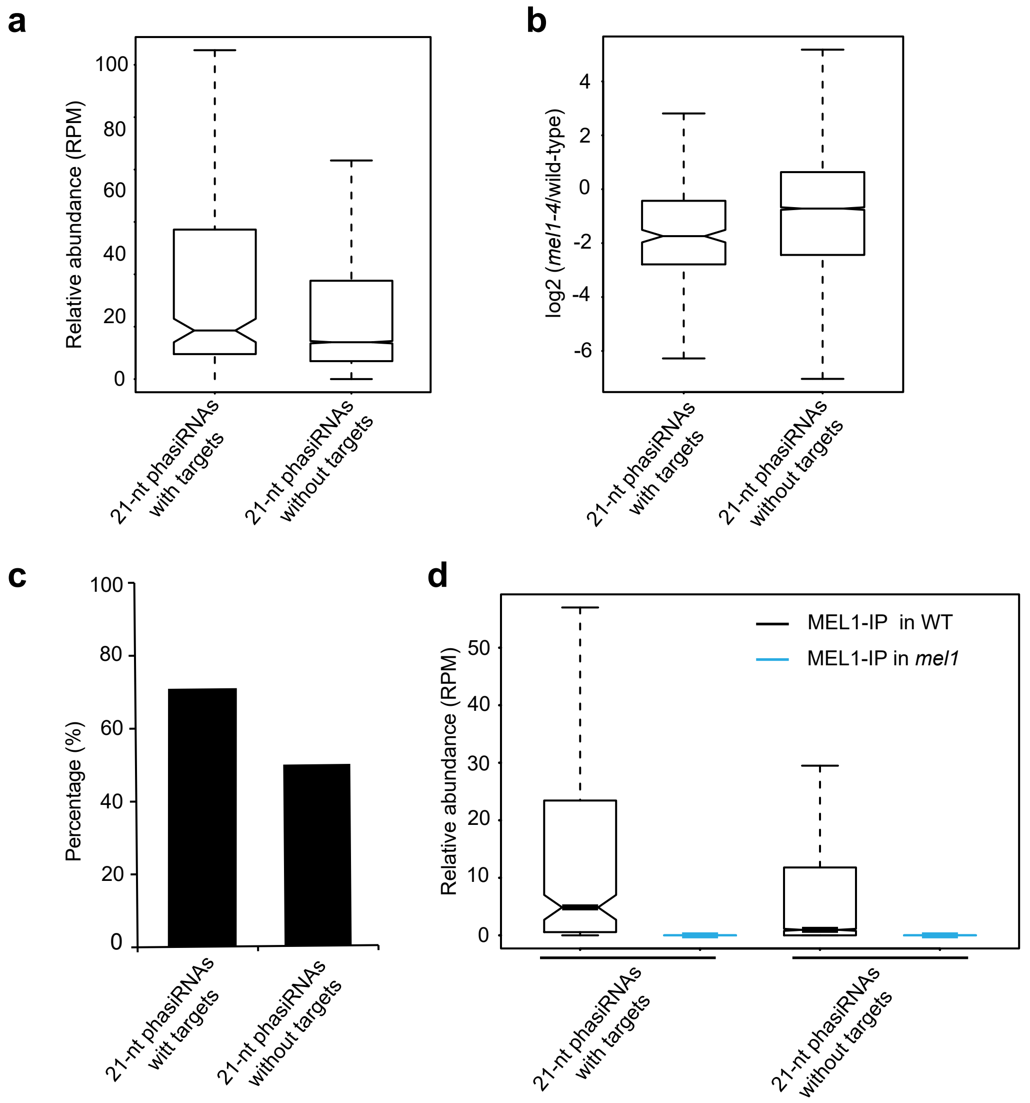

Supplementary Fig. 11. Comparison of the stability of phasiRNAs with and without targets. a, Relative abundances of phasiRNAs with and without cleavage targets in Nipponbare rice plants as determined by sRNA-seq. **b,** Log2 (fold change) of phasiRNA abundance in *mel1-4* relative to its wild-type HHZ. **c,** Percentages of enriched phasiRNAs with and without cleavage targets in MEL1-IP sRNA-seq library. **d,** Relative abundances of phasiRNAs with and without targets in MEL1-IP sRNA-seq library. The central line of the box represents the median while two bounds represent 25% quartile and 75% quartile, respectively. The whisker represents 1.5 × interquartile range of the lower or upper quartile.

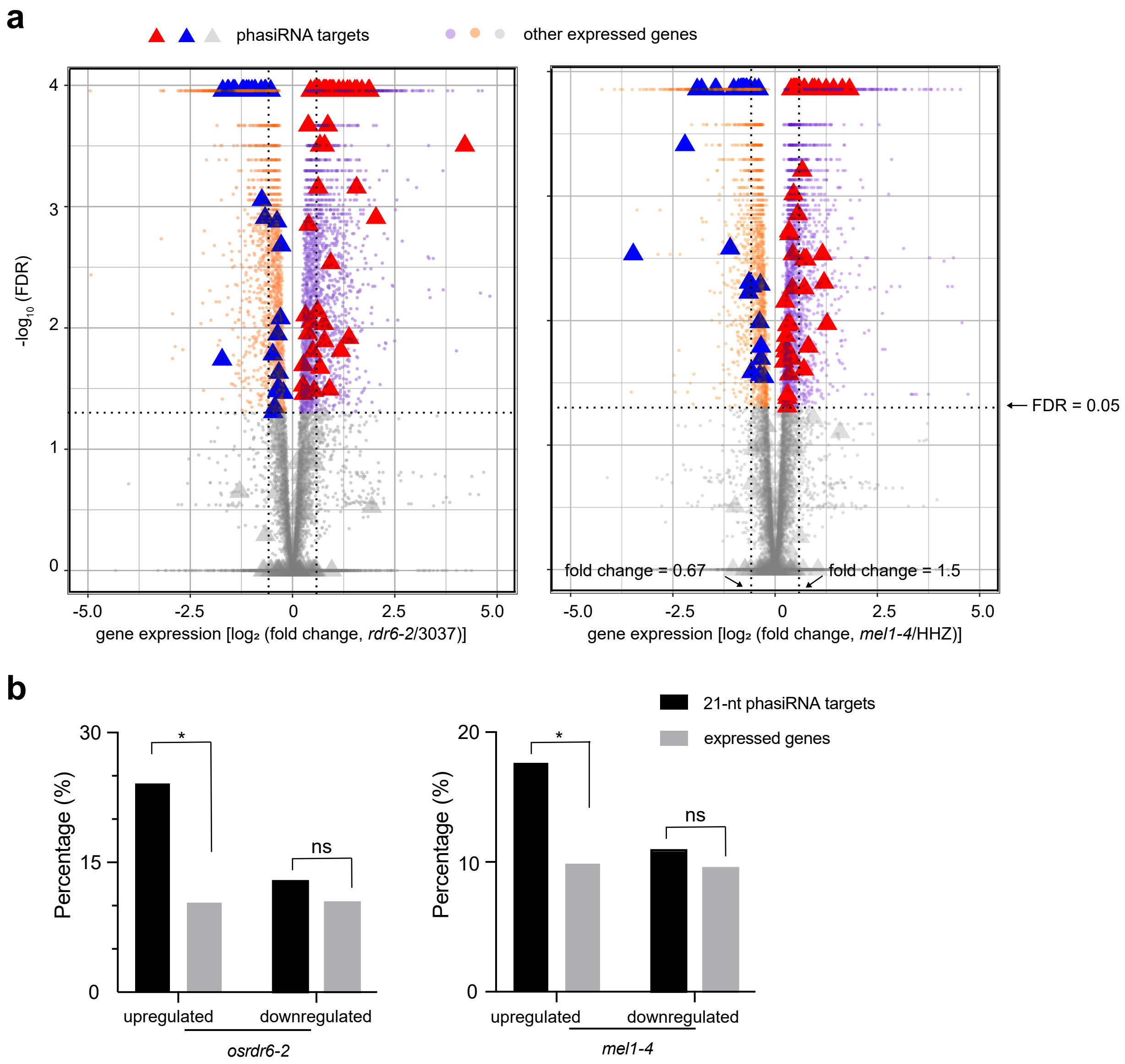

Supplementary Fig. 12. Differential expression of phasiRNA targets in *osrdr6-2* and *mel1-4*. a, Volcano plots showing differential expression of phasiRNA targets and other expressed genes in *osrdr6-2* and *mel1-4*. **b**, Comparison of the percentages of significantly upregulated and downregulated targets in total targets and the percentages of significantly upregulated and downregulated genes in total expressed genes. Fisher's exact test, p-value ≤ 0.05

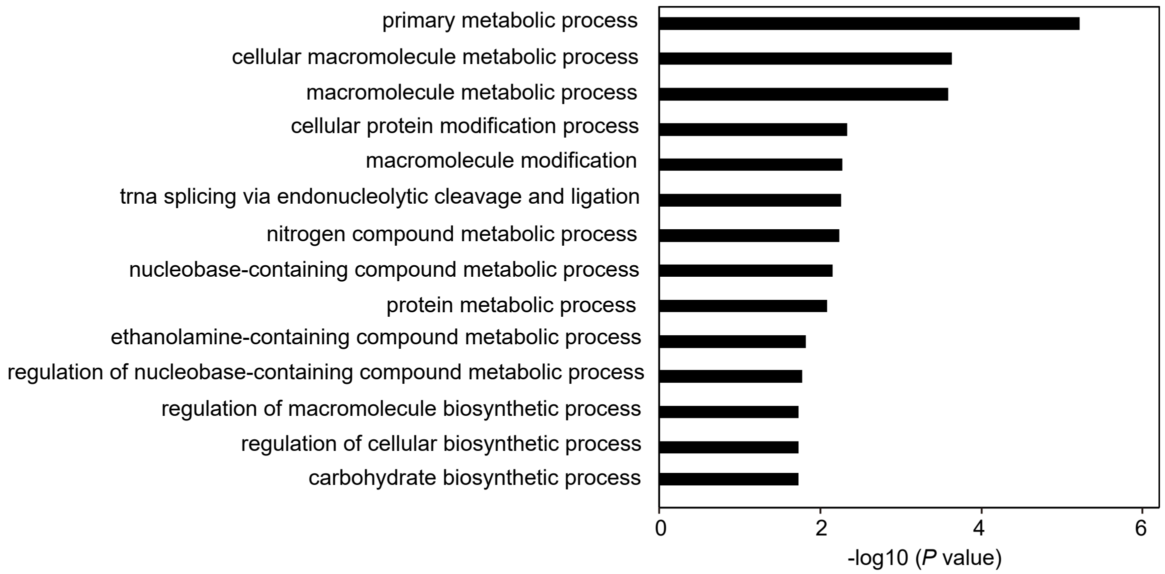

Supplementary Fig. 13. Gene ontology enrichment analysis of the 21-nt phasiRNA targets derepressed in early prophase I meiocytes in *osrdr6-2* or *mel1-4*. GO biological process terms significantly enriched in the targets derepressed in *osrdr6-2* or *mel1-4* are shown. The *P* values were calculated by Fisher's exact test.
