# Supplementary material for: 21-nt phasiRNAs direct target mRNA cleavage in rice male germ cells
Source: Nat Commun. 2020 Oct 15;11:5191. doi: 10.1038/s41467-020-19034-y (PMC7562718; doi:10.1038/s41467-020-19034-y)
Supplement: Supplementary file 3 — Descriptions of Additional Supplementary Files [file 41467_2020_19034_MOESM3_ESM.pdf]

Descriptions of Additional Supplementary Files

Supplementary Data 1

Description: Sequencing datasets generated in this study

Supplementary Data 2

Description: A catalogue of 21- and 24-nt PHAS loci

Supplementary Data 3

Description: Abundances of phasiRNAs in embryos, spikelets and germ cells

Supplementary Data 4

Description: Abundances of phasiRNAs in early prophase I meiocytes of osrdr6-2 and mel1-4 and their respective wild-type plants

Supplementary Data 5

Description: 21-nt phasiRNA targets validated by degradome sequencing

Supplementary Data 6

Description: Degradome counts at cleavage sites of validated 21-nt phasiRNA targets in osrdr6-2, mel1-4 and their respective wild-type plants

Supplemetary Data 7

Description: Expression levels of validated 21-nt phasiRNA targets in the early prophase I meiocytes of osrdr6-2 and mel1-4 and their respective wild-type plants
